# Combining Partially Overlapping Multi-Omics Data in Databases Using Relationship Matrices

**DOI:** 10.3389/fpls.2020.00947

**Published:** 2020-07-14

**Authors:** Deniz Akdemir, Ron Knox, Julio Isidro y Sánchez

**Affiliations:** ^1^Agriculture & Food Science Centre, Animal and Crop Science Division, University College Dublin, Dublin, Ireland; ^2^SCRDC-CRDSW, Swift Current Research and Developmental Centre, Swift Current, SK, Canada; ^3^Centro de Biotecnología y Genómica de Plantas (CBGP, UPM – INIA), Universidad Politécnica de Madrid (UPM) - Instituto Nacional de Investigación y Tecnología Agraria y Alimentaria (INIA), Campus de Montegancedo-UPM, Madrid, Spain

**Keywords:** multi-omics, phenomics, genomic selection, multiple kernel learning, mixed models, covariance estimation, expectation-maximization

## Abstract

Private and public breeding programs, as well as companies and universities, have developed different genomics technologies that have resulted in the generation of unprecedented amounts of sequence data, which bring new challenges in terms of data management, query, and analysis. The magnitude and complexity of these datasets bring new challenges but also an opportunity to use the data available as a whole. Detailed phenotype data, combined with increasing amounts of genomic data, have an enormous potential to accelerate the identification of key traits to improve our understanding of quantitative genetics. Data harmonization enables cross-national and international comparative research, facilitating the extraction of new scientific knowledge. In this paper, we address the complex issue of combining high dimensional and unbalanced omics data. More specifically, we propose a covariance-based method for combining partial datasets in the genotype to phenotype spectrum. This method can be used to combine partially overlapping relationship/covariance matrices. Here, we show with applications that our approach might be advantageous to feature imputation based approaches; we demonstrate how this method can be used in genomic prediction using heterogeneous marker data and also how to combine the data from multiple phenotypic experiments to make inferences about previously unobserved trait relationships. Our results demonstrate that it is possible to harmonize datasets to improve available information across gene-banks, data repositories, or other data resources.

## Introduction

The rapid scientific progress in these genomic approaches is due to the decrease in genotyping costs by the development of next-generation sequencing platforms since 2007 (Mardis, 2008a; Mardis, 2008b). High-throughput instruments are routinely used in laboratories in basic science applications, which has led to the democratization of genome-scale technologies, such as genomic predictions and genome-wide associating mapping studies. Genomic prediction, i.e. predicting an organism’s phenotype using genetic information (Meuwissen et al., 2001), is currently used by many breeding companies because it improves three out of the four factors affecting the breeder equation (Hill and Mackay, 2004). It reduces generation number, improves accuracy of selection, and increases selection intensity for a fixed budget when comparing with marker-assisted selection or phenotypic selection (Heffner et al., 2010; Heffner et al., 2011; de los Campos et al., 2013; Desta and Ortiz, 2014; Juliana et al., 2018). Genomic prediction and selection (GS) are a continuously progressing tool that promises to help meet the human food challenges in the next decades (Crossa et al., 2017). Genome-wide associating mapping studies, which originated in human genetics (Bodmer, 1986; Risch and Merikangas, 1996; Visscher et al., 2017), have also become a routine in plant breeding (Gondro et al., 2013).

The biological data generated in the last few years from this genomic progress have grown exponentially which have led to a high dimensional and unbalanced nature of the “omics” data. Data normally comes in various forms of marker and sequence data: expression, metabolomics, microbiome, classical phenotype, image-based phenotype (Bersanelli et al., 2016). Private and public breeding programs, as well as companies and universities, have developed different genomics technologies that have resulted in the generation of unprecedented levels of sequence data, which bring new challenges in terms of data management, query, and analysis.

It is clear that detailed phenotype data, combined with increasing amounts of genomic data, have an enormous potential to accelerate the identification of key traits to improve our understanding of quantitative genetics (Crossa et al., 2017). Nevertheless, one of the challenges that still need to be addressed is the incompleteness inherent in these data, i.e., several types of genomic/phenotypic information covering only a few of the genotypes under study (Berger et al., 2013). Data harmonization enables cross-national and international comparative research, as well as allows the investigation of whether or not datasets have similarities. In this paper, we address the complex issue of utilizing the high dimensional and unbalanced omics data by combining the relationship information from multiple data sources, and how we can facilitate data integration from interdisciplinary research. The increase of sample size and the improvement of generalizability and validity of research results constitute the most significant benefits of the harmonization process. The ability to effectively harmonize data from different studies and experiments facilitates the rapid extraction of new scientific knowledge.

One way to approach the incompleteness and the disconnection among datasets is to combine the relationship information learned from these datasets. The statistical problem addressed in this paper is the calculation of a combined covariance matrix from incomplete and partially overlapping pieces of covariance matrices that were obtained from independent experiments. We assume that the data is a random sample of partial covariance matrices from a Wishart distribution (Anderson, 2003), then we derive the expectation-maximization algorithm for estimating the parameters of this distribution. According to our best knowledge no such statistical methodology exists, although the proposed method has been inspired by similar methods such as (conditional) iterative proportional fitting for the Gaussian distribution (Cramer, 1998; Cramer, 2000) and a method for combining a pedigree relationship matrix and a genotypic matrix relationship matrix which includes a subset of genotypes from the pedigree-based matrix (Legarra et al., 2009; Christensen et al., 2012) (namely, the H-matrix approach or the related single-step genomic prediction). The applications in this paper are chosen in the area genomic prediction in the case where there is partial genomic and phenotypic information about several populations. However, the statistical method is applicable much beyond the described applications in this article.

The integration of heterogeneous and large omics data constitutes a challenge and an increasing number of scientific studies address this issue. A brief review and classification of some promising statistical approaches are described in Bersanelli et al. (2016). According to this article, our covariance-based method falls in the network-based data integration category (as opposed to non-network based methods such as feature imputation) which include popular methods such as similarity network fusion Wang et al. (2014), weighted multiplex networks Menichetti et al. (2014) both of which can be used to combine several **complete** networks by suitable weighting. The main breakthrough here is that the proposed method in this article can be used to combine several **incomplete but partially overlapping** networks and that the proposed approach is supported theoretically by the maximum likelihood formalization.

## Methods and Materials

### Statistical Methods for Combining Incomplete Data

#### Imputation

The standard method of dealing with heterogeneous data involves the imputation of features (Shrive et al., 2006). If the datasets to be combined overlap over a substantial number of features then the unobserved features in these datasets can be accurately imputed based on some imputation method (Bertsimas et al., 2017).

Imputation step can be done using many different methods: Several popular approaches include Beagle (Browning and Browning, 2016), random forest (Breiman, 2001) imputation, expectation-maximization based imputation (Endelman, 2011), low-rank matrix factorization methods that are implemented in the R package (Hastie and Mazumder, 2015). In addition, parental information can be used to improve imputation accuracies (Browning and Browning, 2009; Nicolazzi et al., 2013; VanRaden et al., 2015; Gonen et al., 2018). In this study, we used the low-rank matrix factorization method in all of the applications which included an imputation step. The selection of this method was due to the computational burden of the other alternatives.

#### Combining Genomic Relationship Matrices

In this section, we describe the Wishart EM-Algorithm for combining partial genetic relationship matrices^1^.

##### Wishart EM-Algorithm for Estimation of a Combined Relationship Matrix From Partial Samples

Let *A* = {*a*_1_, *a*_2_,…,*a_m_*} be the set of partially overlapping subsets of genotypes covering a set of *K* (i.e., $K=\cup_{i=1}^{m}a_{i}$) with total *n* genotypes. Let $G_{a_{1}},G_{a_{2}},\ldots ,G_{a_{m}}$ be the relationship matrices for genotypes in sets *a*_1_, *a*_2_,…,*a_m_* We want to estimate the overall relationship matrix Σ for the *n* genotypes using $G_{a_{1}},G_{a_{2}},\ldots ,G_{a_{m}}.$ Moreover, if we focus on one single relationship matrix $G_{a_{i}}$ we drop the subscript and write *G_a._*

Starting from an initial estimate of the genetic relationship matrix Σ^(0)^ = *ν*Ψ^(0)^, the Wishart EM-Algorithm repeats updating the estimate of the genetic relationship matrix until convergence:

$$\Psi^{\left(t+1\right)}=\frac{1}{\nu m}\underset{a\in A}{\sum }P_{a}\left[\begin{array}{cc} G_{a} & G_{a}(B_{b|a}^{\left(t\right)})\prime \\ B_{b|a}^{\left(t\right)}G_{a} & \nu \Psi_{b|a}^{\left(t\right)}+B_{b|a}^{\left(t\right)}G_{a}(B_{b|a}^{\left(t\right)})\prime \end{array}\right]{P^{\prime }}_{a}$$

where $B_{b|a}^{\left(t\right)}=\Psi_{ab}^{\left(t\right)}{\left(\Psi_{a}^{\left(t\right)}\right)}^{-1},\Psi_{b|a}^{\left(t\right)}=\Psi_{b}^{\left(t\right)}-\Psi_{ab}^{\left(t\right)}{\left(\Psi_{a}^{\left(t\right)}\right)}^{-1}\Psi_{ba}^{\left(t\right)},$ *a* is the set of genotypes in the given partial genomic relationship matrix, *b* is the set difference of *K* and *a*. We assume partitioning of Ψ^(^*^t^*^)^ as

$$\left[\begin{array}{cc} \Psi_{a}^{\left(t\right)} & \Psi_{ab}^{\left(t\right)}\\ \Psi_{ba}^{\left(t\right)} & \Psi_{b}^{\left(t\right)} \end{array}\right]$$

where $\Psi_{a}^{\left(t\right)}$ is the part of matrix that correspond to the genotypes *a*, $\Psi_{b}^{\left(t\right)}$ is the part of matrix that correspond to the genotypes *b*, and $\Psi_{ab}^{\left(t\right)}={\Psi^{\prime }}_{ba}^{\left(t\right)}$ is the part that correspond to the relationship of genotypes in *a* and *b*. The matrices *P_a_* are permutation matrices that put each relationship matrix in the summation in the same order. The superscripts in parenthesis “(*t*)” denote the iteration number. The estimate Ψ^(^*^T^*^)^ at the last iteration converts to the estimated genomic relationship with ${\text{Σ}}^{\left(T\right)}=\nu \Psi^{\left(T\right)}$. Σ^(0)^ is the initial estimate of the relationship of the *n* genotypes that reflects the *a priori* knowledge about the combined relationship.

A weighted version of this algorithm can be obtained replacing *G_a_* in Equation 1 with $G_{a}^{\left(w_{a}\right)}=w_{a}G_{a}+\left(1-w_{a}\right)\nu \Psi_{a}^{\left(t\right)}$ for a vector of weights (*w*_1,_ *w*_2,…,_ *w_m_*)*'*.

Derivation of the Wishart-EM algorithm and its asymptotic errors are given in Supplementary. We note here that the choice of the degrees of freedom parameter *ν* does not affect the estimate of the combined relationship matrix but it has an effect on the asymptotic standard errors. While it is possible to estimate this parameter by maximizing the likelihood function, in practice since we are assuming large samples (many features) go into the calculation of the partial matrices, a large value for *ν* (in the order of the average number of features used in the calculation of the partial matrices) will give reasonable results.

Also, we note that when combining a relationship matrix say *A* with a relationship matrix nested in say *G* it the algorithm can be implemented with Σ^(0)^ = *A* and the single *G* to update it. In this case, the algorithm converges in one iteration and the resulting relationship matrix will be the same as the one that would be obtained by the H-Mat and the related to single-step genomic prediction (Legarra et al., 2009; Christensen et al., 2012) approaches; in other words, our algorithm generalizes their approach to two or more relationship matrices not necessarily nested.

### Materials: Datasets and Experiments

In this section, we describe the datasets and the experiments we have designed to explore and exploit the Wishart EM-Algorithm.

Note that the applications in the main text involve real datasets and validation with such data can only be as good as the ground truth known about the underlying system. We also included several simulation studies in the Supplementary (**Supplementary Applications 1** and **2**) using simulated data to show that the algorithm performs as expected (maximizes the likelihood and provides a “good” estimate of the parameter values) when the ground truth is known.

#### Application 1: Potato Dataset; When Imputation Is Not an Option. Anchoring Independent Pedigree-Based Relationship Matrices Using a Genotypic Relation Matrix

In this application, we demonstrate that genomic relationship matrices can be used to connect several pedigree-based relationship matrices by the Wishart-EM-Algorithm.

The dataset is cited in (Endelman et al., 2018) and is available in the R Package AGHmatrix (Rampazo Amadeu et al., 2016). It consists of the pedigree of 1,138 potato samples, 571 of these genotypes also have data for 3,895 tetraploid markers. The pedigree-based relationship matrix A was calculated with R package AGHmatrix (Rampazo Amadeu et al., 2016) using pedigree records, there were 185 founders (clones with no parent).

**Figure 1:**
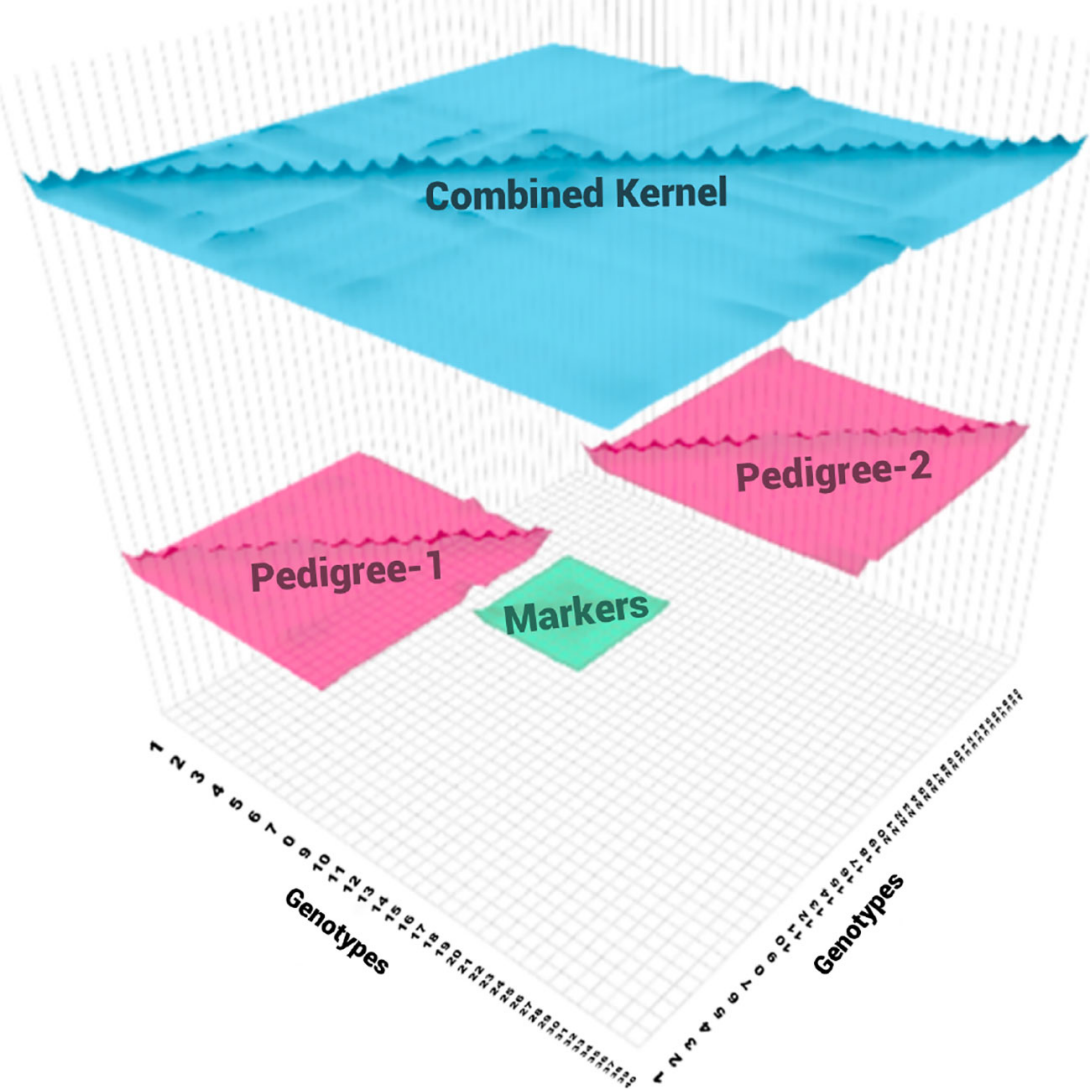
At each replication of the experiment, two non-overlapping pedigree-based relationship matrices (in pink) are selected at random (20 individuals each) from the 571 genotypes. A genomic relationship matrix obtained from a random sample of genotypes (in green), half from the genotypes in the first pedigree (10) and half from the genotypes from the second pedigree (10). These three relationship matrices were combined to get a combined relationship matrix (in blue).

The application experiment was structured as follows:

1. A random sample (*Nped*) of two non-overlapping pedigree-based relationship matrix *Nped* ∈ {100, 150, 250} were selected. This means, there is no information in common between pedigree.
2. A random sample (*Ngeno*) from half of the genotypes from each pedigree was selected to create a genotypic relationship matrix. This means that in each *Ngeno* ∈{20,40,80} half of the genotypes come from one pedigree and the other half from the other. This allows us to have partially overlapping data to create a combined relationship matrix.
3. These genetic relationship matrices were combined to get a combined genetic relationship matrix (See **Figure 1**).

This combined relationship matrix was compared to the pedigree-based relationship matrix of the corresponding genotypes using mean squared errors and Pearson’s correlations. These correlations and the mean squared errors were calculated only using the unobserved (validation) part of the combined relationship matrix. This experiment was repeated 30 times for each *Ngeno*, *Nped* pair.

#### Application 2: Rice Dataset. Combining Independent Low-Density Marker Datasets

Rice dataset was downloaded from www.ricediversity.org. After curation, the marker dataset consisted of 1,127 genotypes observed for 387,161 markers. We treat the totality of information as the ground truth, i.e., we assume that the true genomic relationship for the 1,127 genotypes is characterized by the 387,161 markers. The purpose of this application is to demonstrate that we can make inferences about the assumed true genomic relationship matrix by observing several smaller heterogeneous subsets of the available. This involves inferring a common estimate for the relationships that are already observed and producing estimates for relationships that haven’t been observed. **Supplementary Figure S5** demonstrate this experiment pictorially.

In each instance of the experiment, *N_kernel_* ∈ {3, 5, 10, 20, 40, 80} marker datasets with 200 genotypes and 2,000 markers were created by randomly sampling the genotypes and markers in each genotype file. These datasets were combined using the Wishart EM-Algorithm and also by imputation to give two genomic relationship matrices. For the totality of genotypes in these combined datasets, we also randomly sampled 2,000, 5,000, or 10,000 markers, and calculated the genomic relationships based on these marker subsets. All of these genomic relationship matrices were compared with the corresponding elements of the relationship matrix based on the entire genomic data by calculating the mean squared error and correlation between the upper diagonal elements including the diagonals. This experiment was replicated 20 times. Application results are showed in **Figure 8**.

#### Application 3: Wheat Data at Triticeae Toolbox. Combining Genomic Datasets to Use in Genomic Prediction

This application involves estimating breeding values for seven economically important traits for 9,102 wheat lines obtained by combining 16 publicly available genotypic datasets. The genotypic and phenotypic data were downloaded from the Triticeae toolbox database. Each of the marker datasets was pre-processed to produce the corresponding genomic relationship matrices. **Table 1** and **Supplementary Figure S7** describes the phenotypic records and number of distinct genotypes for each trait.

**Table 1:** Marker datasets from Triticeae Toolbox: Labels and names for the datasets, number of genotypes and markers in each of the selected 16 genotypic datasets.

Label	Data	# Genotypes	# Markers
d1	2012_SRWW_ElitePanel	276	90,782
d2	2014_HAPMAP	53	180,198
d3	2014_SRWW_YNVP	307	109,073
d4	2014_TCAPABBSRWMID	365	100,340
d5	CornellMaster_2013	1,128	18,846
d6	Dart_NebDuplicates_2010	278	1,970
d7	HWWAMP_2013	288	32,288
d8	HWWAMP_2014	311	265,551
d9	NSGC9k_spring	2,196	5,303
d10	NSGC9k_winter	1,674	5,010
d11	TCAP90k_HWWAMP_SPRN	20	16,842
d12	TCAP90k_LeafRust	339	24,610
d13	TCAP90k_NAMparents	60	25,851
d14	TCAP90k_SpringAm	248	24,343
d15	TCAP90k_SWW	317	24,978
d16	WWDP9k	2,258	6,232

**Figure 2:**
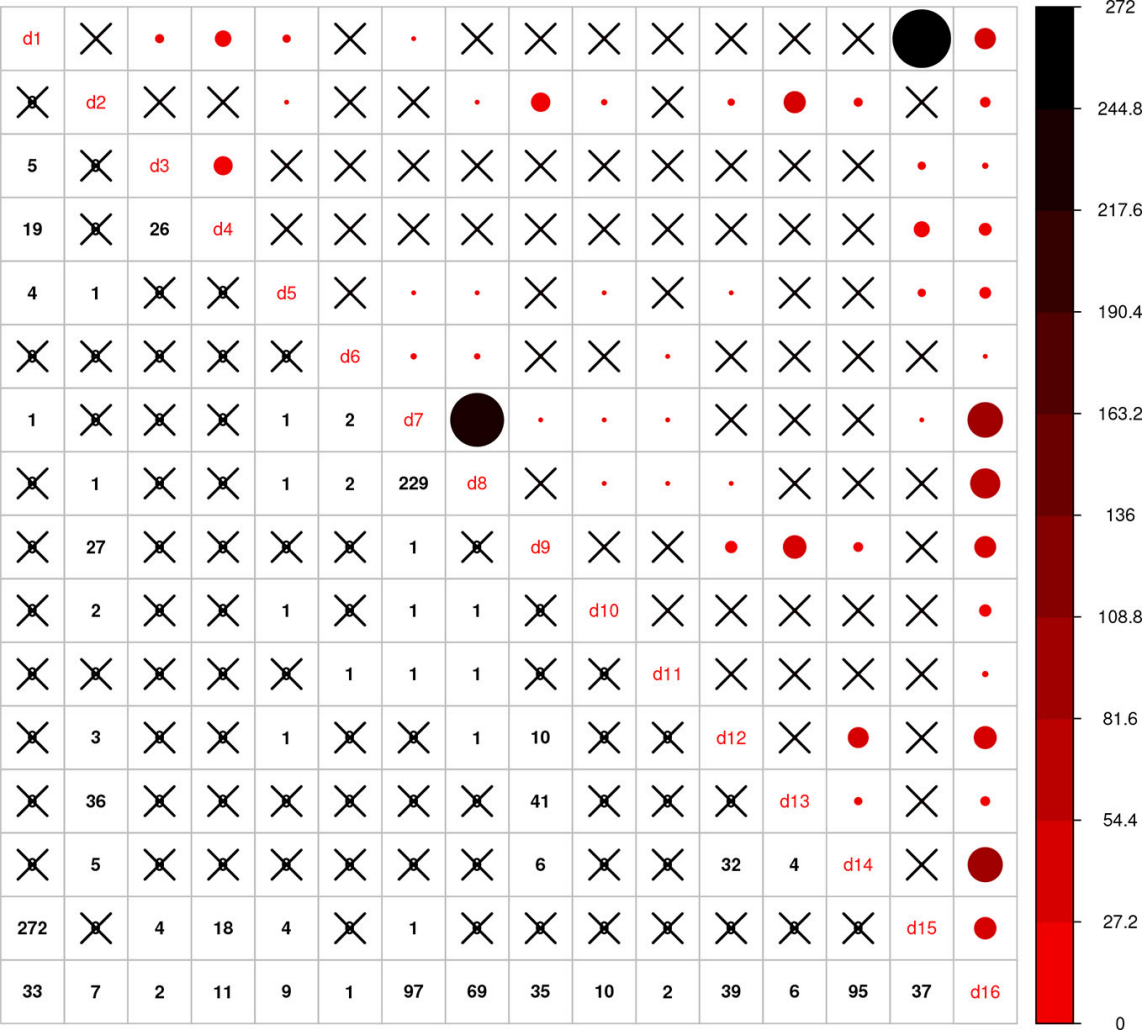
Intersection of genotypes among 16 genotypic experiments. The number of common genotypes among the 16 genotypic datasets are given on the lower diagonal, no intersection is marked by “X.” Upper diagonal of the figure gives a graphical representation of the same, larger circles represent higher number of intersections.

**Figure 3:**
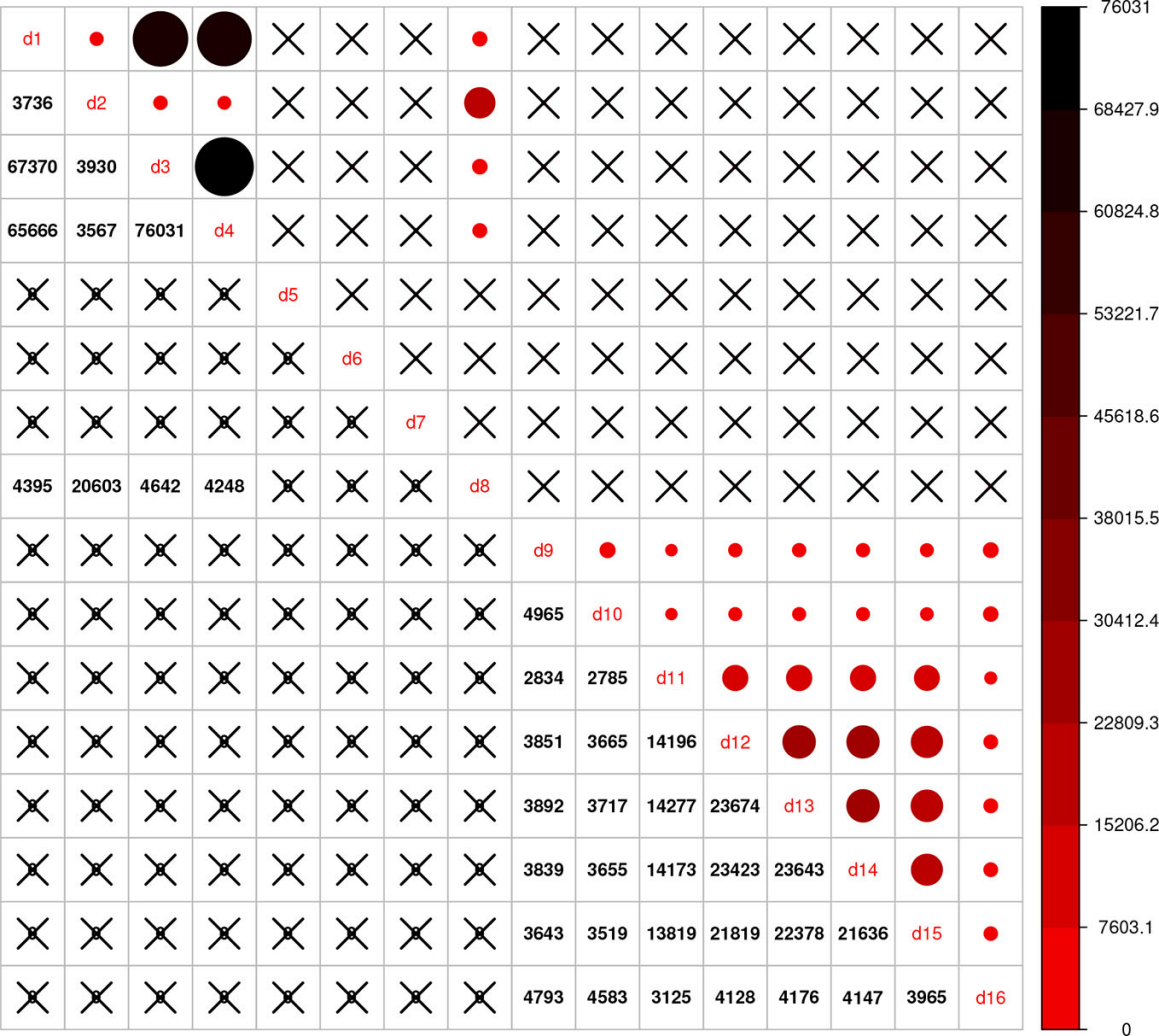
Intersection of markers among 16 genotypic experiments. The number of common markers among the 16 genotypic datasets are given on the lower diagonal, no intersection is marked by “X.” Upper diagonal of the figure gives a graphical representation of the same thing.

**Figure 4:**
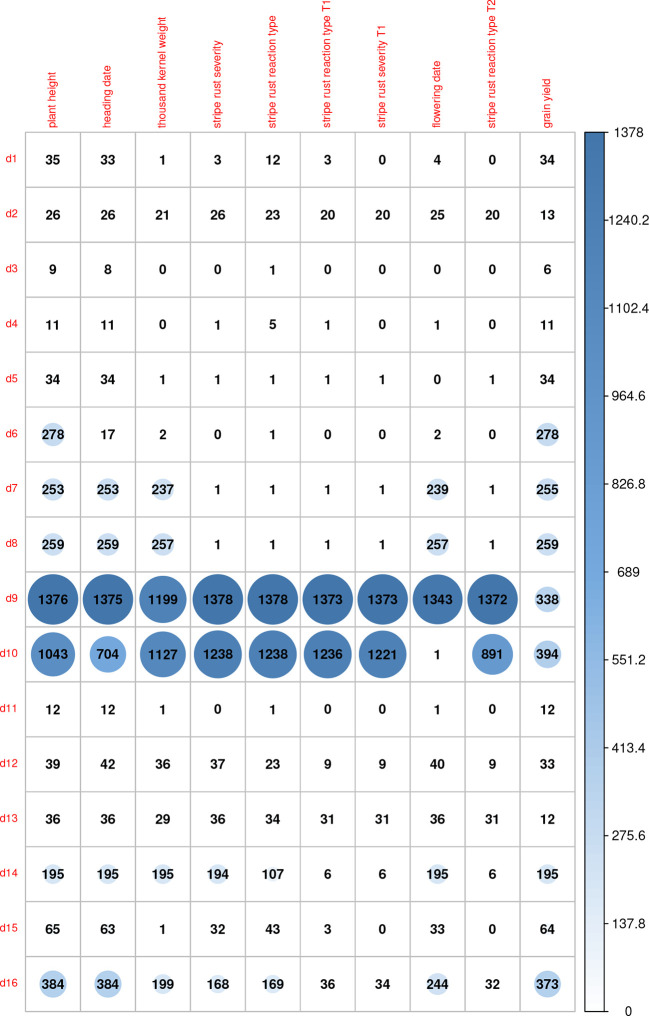
Availability of phenotypic data for the genotypes in 16 genotypic datasets for 10 traits. Here we indicated the traits with most phenotypic records for the genotypes in the 16 genotypic datasets. Plant height, grain yield, and heading time are the most measured trait across all the environments. Some trials have few measures. This graph shows the unbalanced and the need for harmonization of datasets.

Using the combined relationship matrix we can build genomic prediction models. To test the performance of predictions based on the combined relationship matrix, we formulated two cross-validation scenarios. The common genotypes among the 16 genotypic experiments are shown in **Figure 2** and the common markers among genotypic experiments in **Figure 3**. The availability of the phenotypic data for all the datasets are showed in **Figure 4**.

- **Cross-validation scenario 1**
  The first scenario involved a 10 fold cross-validation based on a random split of the data. For each trait, the available genotypes were split into 10 random folds. The GEBVs for each fold was estimated from a mixed model (see **Supplementary Section 5.4** for a description of this model) that was trained on the phenotypes available for the remaining genotypes. The accuracy of the predictions was evaluated by calculating the correlations between the GEBVs and the observed trait values.
- **Cross-validation scenario 2**
  Here, we performed a leave one dataset out cross-validation. i.e. we leave out the phenotypic values for the traits of the associated genotypes in one of the 16 genomic datasets and then estimate the trait values of those genotypes based on a mixed trained model. The training population was built on the remaining genotypes and phenotypic information after leaving the phenotypic records out. This scenario was used for each trait, and the accuracies were evaluated by calculating the correlations between the estimated and the observed trait values within each dataset.

#### Application 4: Maize Data—Genomics and Transcriptomics for Genomic Prediction

In this application, we look into the effects of marker density and data size overlapping on genome-wide relationship matrix and genomic prediction accuracies using a multi-omics data that includes 332,177 genotypic markers and 31,237 feature transcriptomics. The phenotypes used in this application are yield, height, and flowering time from 388 maize lines. More information about the dataset and how it was curated can be found in Azodi et al. (2020).

The aim of this application was i) to study the effect of the number of genotypes common across different populations on the genomic prediction accuracies and ii) to evaluate the effect of the number of genotypes common across different populations and the marker density on the accuracy of predicting unobserved genomic relationships.

To accomplish the first objective we perform the following steps in a cross-validation experiment which was repeated 50 times.

**Figure 5:**
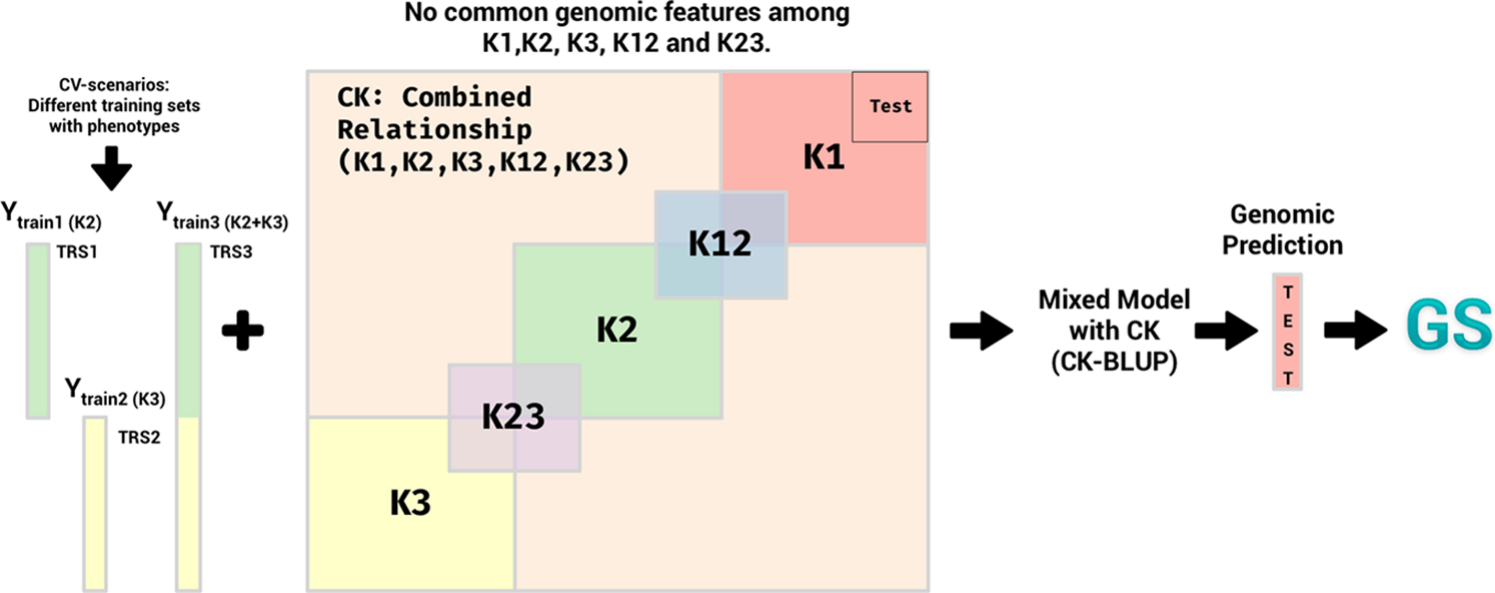
Application 5: Design of the experiment for Maize Data–Genomics and Transcriptomics for Genomic Prediction. In this figure, we represent the study of the effect of the number of genotypes common across different populations on the genomic prediction accuracies (First objective of Application 4. The second objective is similar but with minor changes). Here, genotypes were randomly partitioned into three groups without common genotypes. The relationship matrices for these groups are K1, K2, and K3 and are genomic relationship matrices by marker or transcriptomics data. K12, K23 are genomic relationship matrices by marker or transcriptomics data that connect K1, K2, and K3. The combined relationship is denoted by CK. In the objective 1, there are two different cross-validation scenarios, i) When K1, K2, K3 are marker and K12, K23 are transcriptomics relationship matrices, and ii) when K12, K23 are marker and K1, K2, and K3 are transcriptomics relationship matrices. In all scenarios, K12 or K23 cover 20, 40, and 50% of genotypes in K1, K2, and K3. We performed different training population scenarios (TRS1, TRS2, and TRS3; each TRS color matches the relationship matrices colors in K2, K3) with different relationship matrices to predict the Test population. A random subset of genotypes in K1 that are not in K12 are identified as the Test set population.

1. First the genotypes in the dataset were randomly partitioned into three groups with 128, 130, and 130 individuals in them. These groups do not have common genotypes. We named the relationship matrices for these different sets of genotypes as K1, K2, and K3. After this, a percentage (20, 40, 50%) of genotypes from K1 and the same percentage of genotypes from K2 were randomly selected and the relationship matrix for these genotypes is denoted by K12. Similarly, the same percentage of genotypes as above from K2 and the same percentage of genotypes from K3 were randomly selected and the relationship matrix for these genotypes is denoted by K23. Additionally, a random subset of genotypes in K1 that are not in K12 are identified as the Test (validation) genotypes (see **Figure 5** for the split of genotypes into these sets).
2. Two different combined genomic relationship matrices are calculated using two different scenarios. In scenario 1, we assume K1, K2, and K3 are relationship matrices obtained from different partitions of the whole markers dataset divided into three groups. On the other hand, K12 and K23 are obtained from different partitions of the transcriptions divided randomly into two. Since the majority of the individuals have markers we denote this scenario as “Geno.” In scenario 2, the method is the same but we replace the role of genotypic markers and transcriptomics. In this case, K1, K2, and K3 are relationship matrices from transcriptomics and K12 and K23 are obtained from genomic markers.
3. We used three different training population (TRS) methods. The first training population only uses individuals in K2 as training (Train1, TRS1), the second training population only uses the genotypes in K3 as training (Train2, TRS2). Finally, the union of these individuals makes up what we call Train3 or TRS3 (**Figure 5**).
4. CK-BLUP models were trained using the phenotypes from three different training sets and using the two combined relationship matrices. Also, a G-BLUP model using the full genetic information (388 genotypes and 332,177 markers), a G-BLUP model using full transcriptomic information (388 genotypes and 31,237 transcriptomes), and a multiple-kernel mixed-effects model which combined these two matrices were build using the same three training sets.
5. Each model is used to predict the individuals in the test sets and the predictions were compared to the available phenotypic values using correlation as the agreement measure.

To accomplish the second objective, we devised a similar cross-validation experiment as the first objective with the following changes.

- We used only the genomic marker data (no transcriptomics), i.e., K1, K2, K3, K12, K23 are all marker-based genomic relationship matrices.
- The number of markers for estimating the partial relationship matrices K1, K2, K3, K12, K23 were changed between 1,000 and 40,000 with no common markers across datasets.
- The number of overlap between K12 and K1 (also K12 and K2), similarly the overlap between K23 and K2 (also K23 and K3) is changed between 10 and 60.
- The accuracy (Coefficient of determination *R*^2^) of estimating the unobserved genomic relationships were calculated after estimating the combined relationship matrix and comparing it to the corresponding elements of the marker-based relationship matrix that was obtained using all 388 genotypes and all of the 332,177 markers (**Figure 12**).

#### Application 5: Wheat Data at Triticeae Toolbox. Combining Phenotypic Experiments

**Figure 6:**
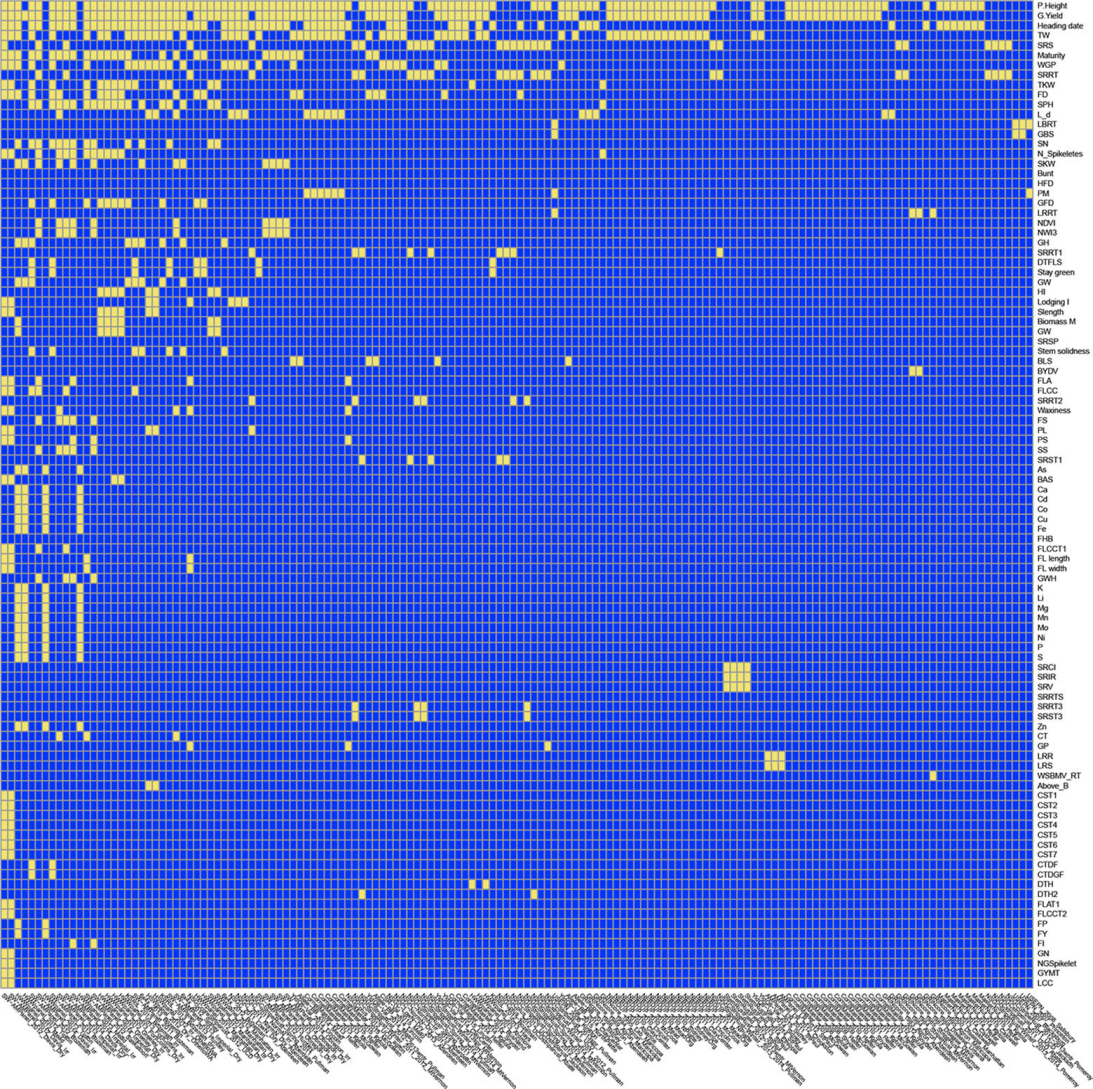
Application 5: Availability of data in 144 phenotypic trials and 95 traits at Triticeae Toolbox for wheat. Yellow shows available data, blue shows unavailable data. The traits and trials are sorted based on availability. Plant height was the most commonly observed trait followed by grain yield.

The Wishart EM-Algorithm can also be used to combine correlation matrices^2^ obtained from independent phenotypic experiments. One hundred forty-four phenotypic experiments involving 95 traits in total were selected from 2,084 trials and 216 traits available at the Triticeae Toolbox. In this filtered set of trials, each trial and trait combination had at least 100 observations and two traits. Furthermore, the percentage of missingness in these datasets was at most 70%. The mean and the median of the number of traits in these trials were 5.9 and 4 correspondingly (See **Figure 6** and **Supplementary Figure S6**).

The correlation matrix for the traits in each trial was calculated and then combined using the Wishart EM-Algorithm. The resulting covariance matrix was used in learning a directed acyclic graph (DAG) using the qgraph R Package (Epskamp et al., 2012).

Another application that involved combining the phenotypic correlation matrices from oat (78 correlation matrices), barley (143 correlation matrices), and wheat (144 correlation matrices) datasets downloaded and selected in a similar way as above were combined to obtain the DAG involving 196 traits in the Supplementary (**Supplementary Application 6.1**).

## Results

### Application 1: When Imputation Is Not an Option: Anchoring Independent Pedigree-Based Relationship Matrices Using a Genotypic Relation Matrix—Potato Data

**Figure 7:**
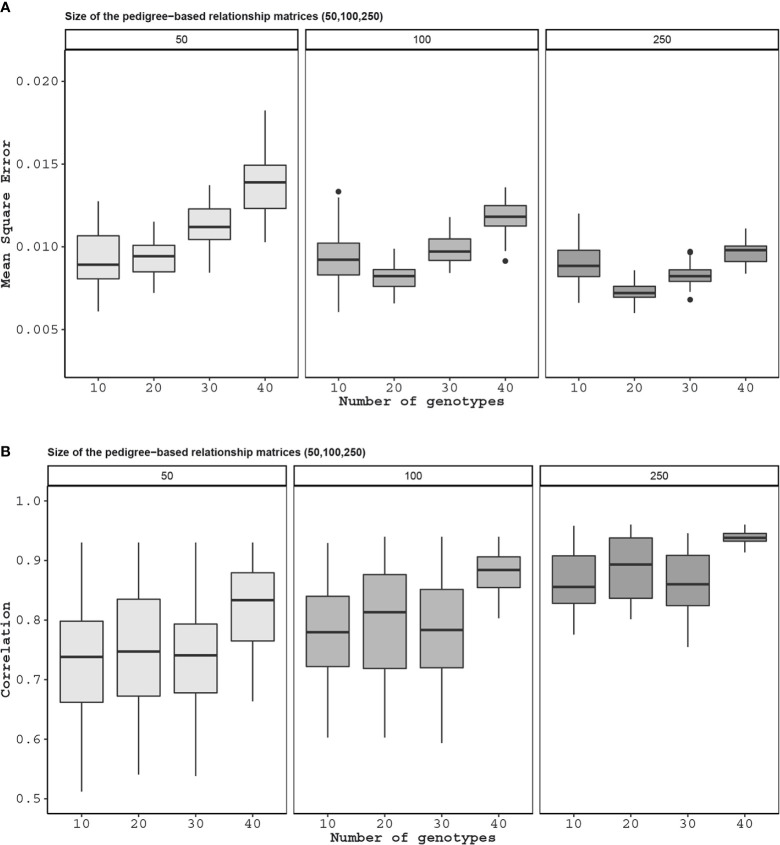
Application 1: For this application, the pedigree was split into two pieces although there is only one pedigree. The number on top of the figure is the number of genotypes in each pedigree. Here, we do not know the relationship between one of the pedigrees to the other. To learn the relationship between the two, we take 10, 20, 30, and 40 individuals from each group and genotype them by next-generation sequencing. The mean square errors **(A)** and correlation values **(B)** are the comparison between the two non-overlapping pedigree-based relationship matrices from each sample size, i.e. 100 individuals from 50 pedigree-based one, and the combined relationship matrix that had 10, 20, 30, and 40 genotypes in each of the pedigrees.

**Figure 7** shows the correlation and mean squared error (MSE) results as either of the sizes of the pedigree matrices and the number of genotypes in the genomic relationship matrices increases. The MSE results for these experiments ranged from 0.004 to 0.017 with a mean of 0.009, and the correlation values ranged from 0.52 to 0.94 with a mean of 0.78.

### Application 2: Rice Dataset. Combining Independent Low-Density Marker Datasets

**Figure 8:**
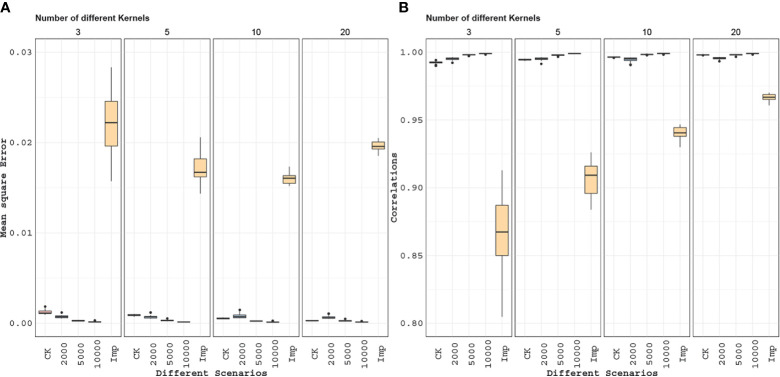
Application 2: Here, we compare marker imputation with our combining relationships matrices approach. Mean square errors **(A)** and correlations values **(B)** between the estimated and full genomic relationship matrices are displayed in the boxplots above. The combined relationship matrix (CK) predicts the structure of the population more accurately than the relationship matrix obtained by imputing the genomic features. Besides, when we compare the combined relationship matrix obtained from partially overlapping marker data sets to the relationship matrices obtained from data with a fixed number of markers (2,000, 5,000, 10,000) observed on all individuals we see that combined kernel can be more accurate when the number of partially overlapping marker data sets is large.

The MSE and correlation results for this experiment are given in **Figure 8**. In general, as the number of independent datasets increases the accuracy of all of the methods/scenarios increases (decreasing MSEs and increasing correlations). In general, the accuracy of the Wishart EM-algorithm in terms of MSEs ranged from 0.0003 to 0.028 with a mean value of 0.0007. The accuracies measured in correlation ranged from 0.989 to 0.998 with a mean value of 0.995. For the imputation based method MSEs ranged from 0.014 to 0.028 (mean 0.019) and the correlations ranged from 0.805 to 0.970 (mean 0.920).

**Figure 9:**
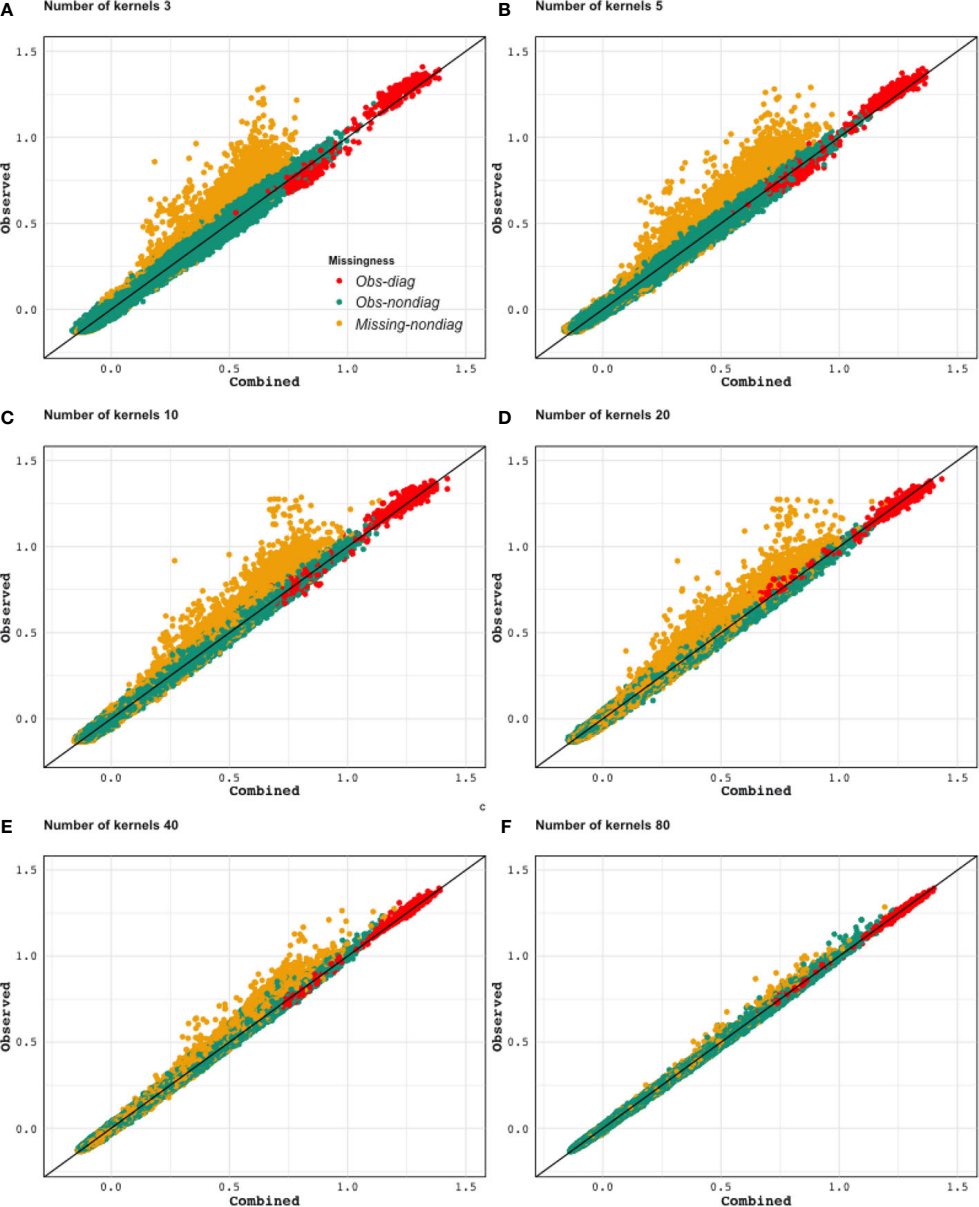
Application 2: Scatter plot of the lower triangular elements of the combined kernel [from 3 **(A)** to 80 **(F)** kernels] against the kernel calculated from all available markers (Observed). As the number of incomplete datasets increases, both observed and unobserved parts of the relationship can be estimated more precisely. Yellow dots: Genotype relationships that are inferred (not observed in any of the partial relationship matrices that are being combined). Red dots: Diagonal elements of the genotypic relationship matrix. Green dots: Genotype relationships that were observed in one or more of the partial relationship matrices.

**Figure 9** displays the scatter plot of full genomic relationship matrix (obtained using all 387,161 markers) against the one obtained by combining a sample of partial relationship matrices (200 randomly selected genotypes and 2,000 randomly selected markers each) over varying numbers of samples (3, 5, 10, 20, 40, and 80 partial relationship matrices). Observed parts (observed-diagonal and observed non-diagonal) of the genomic relationship matrix can be predicted with high accuracy and no bias. As the sample size increase, the estimates get closer to the one obtained using all of the data. We observe that the estimates of the unobserved parts of the relationship are biased towards zero but his bias quickly decreases as the sample size increases.

### Application 3: Wheat Data at Triticeae Toolbox. Combining Genomic Datasets to Use in Genomic Prediction

**Figure 10:**
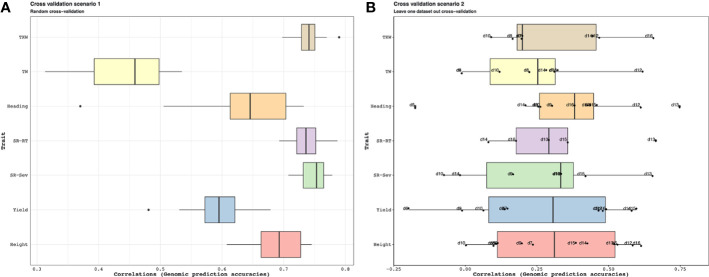
Application 3: Cross-validation scenario 1 is showed in a. For each trait, the available genotypes were split into 10 random folds. The GEBVs for each fold was estimated from a mixed model (See **Supplementary Section 5.4** for a description of this model) that was trained on the phenotypes available for the remaining genotypes. Cross-validation scenario 2 is shown in b. Genotypes in each genotypic data are the test and the remaining genotypes are training. In this case, each data that was predicted was also marked on the boxplots. For instance, for plant height, we can predict the phenotypes for the genotypes in d16 with high accuracy when we use the phenotypes of the remaining genotypes as training dataset; on the other hand, we have about zero accuracies when we try to estimate the phenotypes for the genotypes in d10. The accuracy of the predictions under both scenarios was evaluated by calculating the correlations between the GEBVs and the observed trait values. For each of the traits in this analysis, the accuracies in **(A)** are higher on average than the accuracies in **(B)** pointing to the difficulty of genomic prediction over heterogeneous populations.

The results summarized by **Figure 10** indicate that when a random sample of genotypes are selected for the test population, the accuracy of the genomic predictions using the combined genomic relationship matrix can be high (Cross-validation scenario 1). Average accuracy for estimating plant height was about 0.68, and for yield 0.58. The lowest accuracy values were for test weight with a mean value of 0.48. The performance decreases significantly across population predictions (Cross-validation scenario 2). Some populations showed low prediction accuracies such as d5, d6, and d7, but other as d12 and d16 showed high predictability. Average accuracy for estimating plant height was about 0.30, for yield 0.28.

### Application 4: Maize Data—Genomics and Transcriptomics for Genomic Prediction

**Figure 11:**
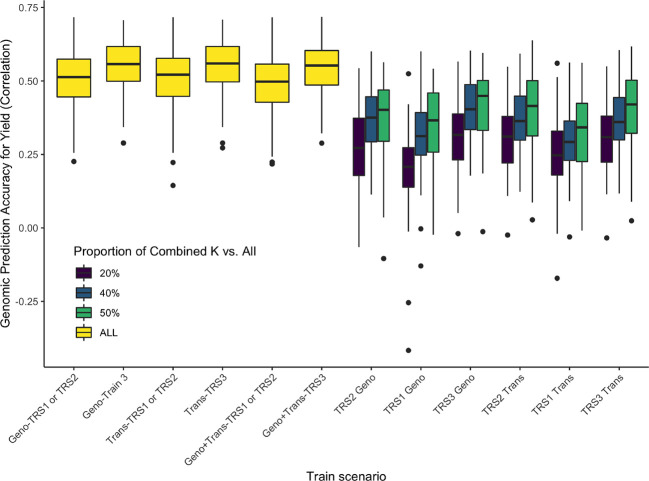
Application 4, Scenario 1: Accuracies (measured in terms of the correlation between the predicted and observed values in the test set shown on the vertical axis) of several models (horizontal axis) using full data and combined relationship matrices from partial observations for estimating yield. The different colors represent the availability of data: yellow bar plots correspond to full data models, the color of the other bar plots represents the percentage of overlap between K1 and K12 (and similarly K2 and K12, K2, and K23, K3, and K23). On the horizontal axis, the combined relationship models are labeled as Geno (or Trans) if K1, K2, K3 are marker (transcriptomics) based, and K12, K23 are transcriptomics (marker) based relationship matrices. In addition, for these models, the training population that the model was trained on is represented as K2, K3, and K2+K3. The training populations were labeled as TRS1, TRS2, TRS3 (**Figure 5**). In these models, the label Geno (or Trans) refers to a G-BLUP model that uses only the marker (transcriptomics) based relationship matrix, Geno+Trans refers to a multi-kernel mixed model that incorporates both of these relationship matrices.

**Figure 12:**
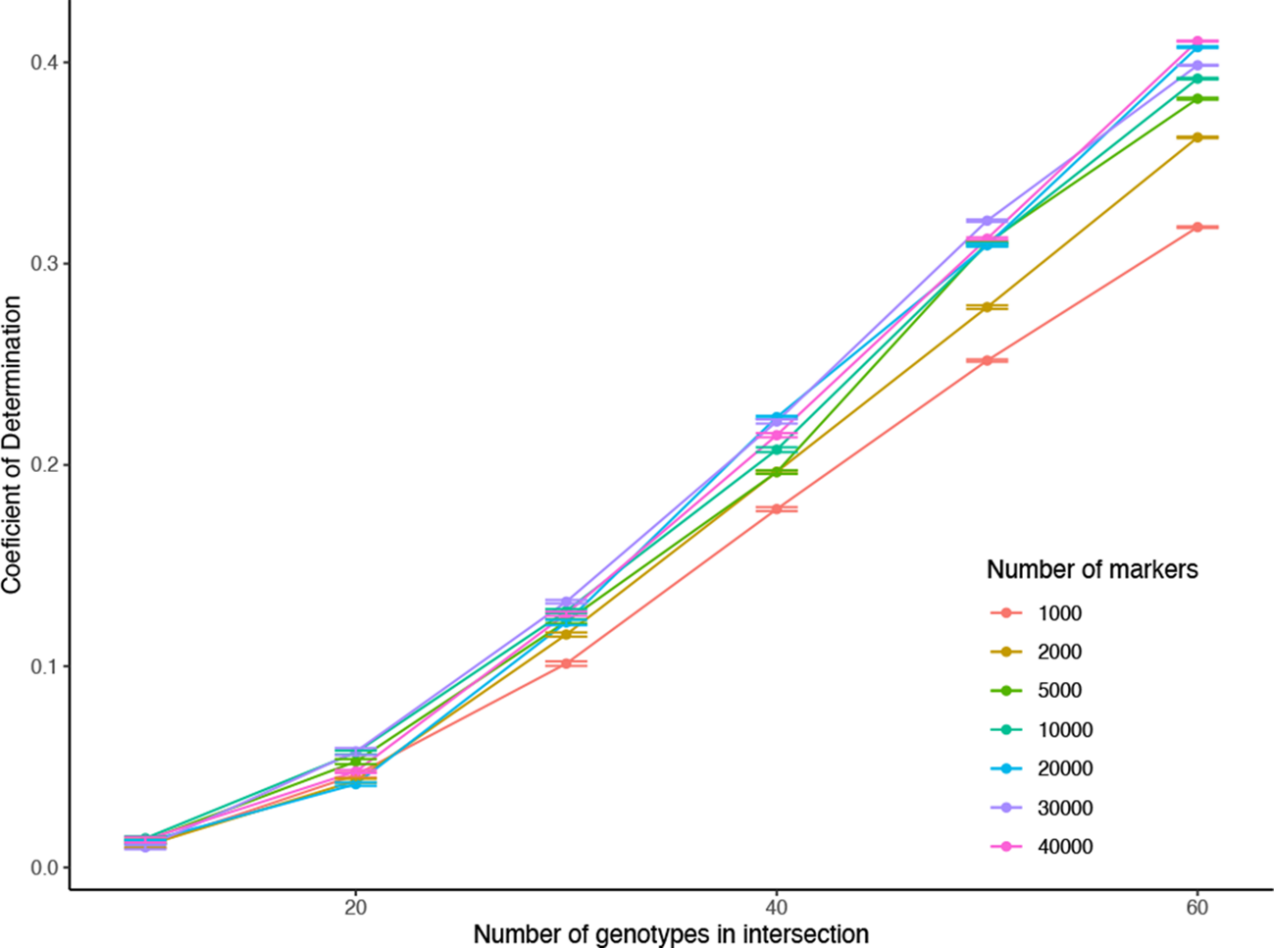
Application 4, Scenario 2: Accuracy of estimating the for changing the number of genotypes in K12, K23 (different colored lines), and also for changing numbers of markers used in calculating each of the relationship matrices K1, K2, K3, K12, and K23 (horizontal axis). The vertical axis shows the *R*^2^ values obtained by taking the square of the accuracies measured by the correlation between the validation part of completed relationship matrices and corresponding elements in the relationship matrix obtained all of the available genotypes and markers.

**Figures 11** and **12** show comparisons of full data accuracies *vs.* partial relationship data. As expected, as the number of common genotypes increases there is a decrease on the differences to the full data. Our results show that up to 80% of the genomic prediction accuracy can be recovered using 50% overlap partial relationship data (**Figure 11**). The results in **Figure 11** point to the feasibility of the application of the CK-BLUP approach when only partial data is available. With the CK approach, we can stitch several genetic relationship matrices together to extend genomic predictions although no genomic features are common between the training and test sets. Besides, as the amount of connection between the different genotypic relationship matrices increases the accuracy also improves. For example, as we increase the number of genotypes in K12, and K23, the accuracy of predictions of the unobserved relationships improve as seen in **Figure 12**. The number of markers seems to have a secondary effect that is more pronounced when the number of genotypes in K12 and K23 becomes larger.

### Application 5: Wheat Data at Triticeae Toolbox—Combining Phenotypic Experiments

**Figure 13:**
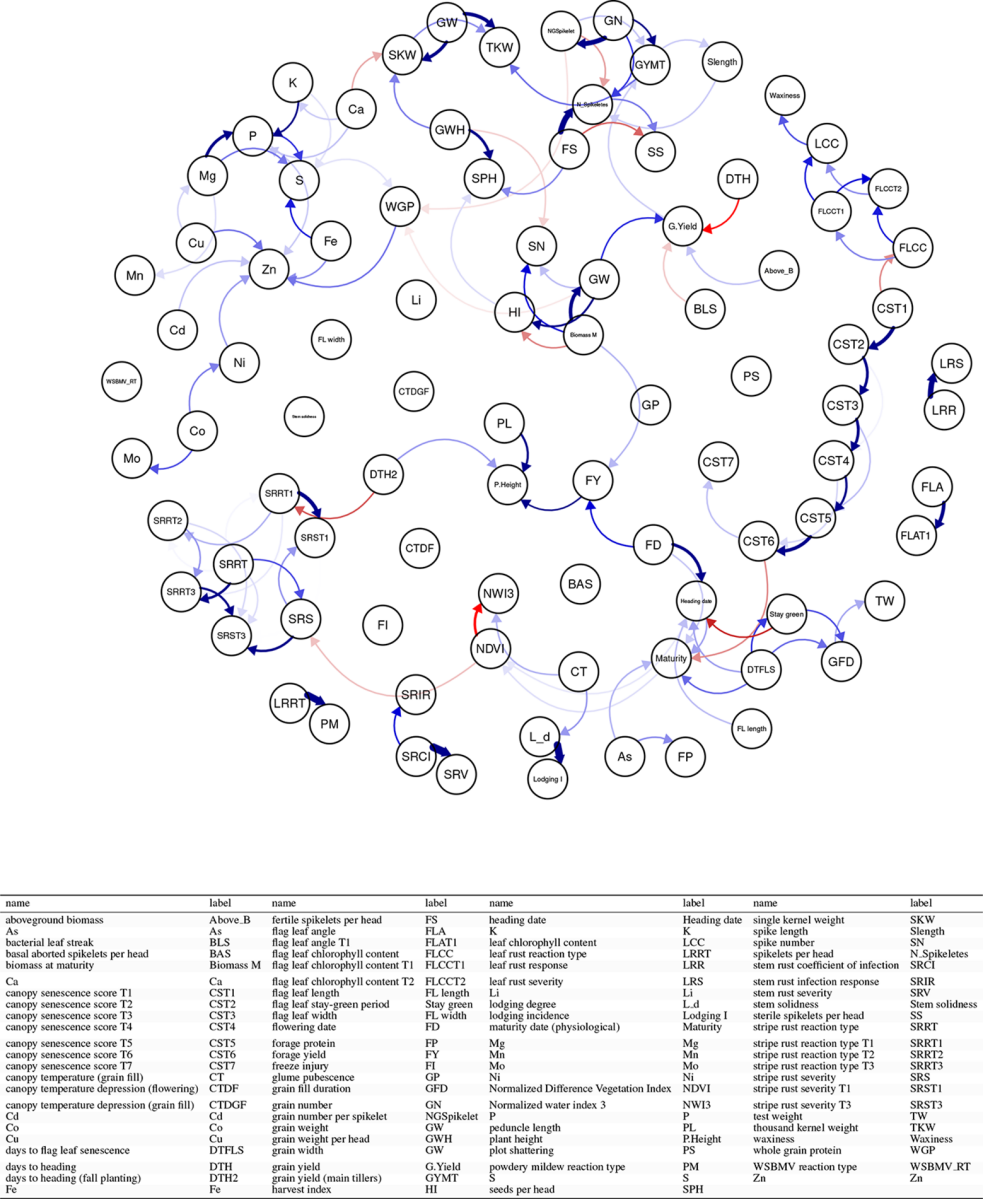
Application 5: Combining the phenotypic correlation matrices from 144 wheat datasets covering 95 traits and illustrating the relationships between traits using the directed acyclic graph as a tool to explore the underlying relationships. Each node represents a trait and each edge represents a correlation between two traits. Blue edges indicate positive correlations, red edges indicate negative correlations, and the width and color of the edges correspond to the absolute value of the correlations: the higher the correlation, the thicker and more saturated is the edge.

In this application, we combined correlation matrices obtained from independent phenotypic experiments. **Figures 13** and **S3** displayed the correlation matrix for the traits in a directed acyclic graph (DAG) and a heatmap, respectively. In **Figure 13** each node represents a trait and each edge represents a correlation between two traits. One of the strengths of this representation is that you can elucidate the correlation between traits that you did not measure in your experiment. For example, among all the traits, grain width (GW) and above-ground biomass (Above_bm) are positively correlated (blue arrows) with grain yield. In turn, GW is highly positively correlated with biomass at maturity (Biomass M) but negatively correlated with harvest index (HI). Negative correlations (red) can also be observed among traits. Traditional inverse correlations such as protein (WGP) and GW can be also observed.

Combining datasets by correlation matrices also help to group traits. **Figure S3** shows two groups of positively correlated traits. The traits in these two groups are positively correlated within the group but negatively correlated with traits in other groups. For instance, we see that yield-related traits such as grain yield, grain weight, or harvest index, are positively correlated. On the other hand, these traits are negatively correlated with disease-related traits such as bacterial leaf streak, stripe rust traits, and also with quality traits such as protein and nutrient content.

## Discussion and Conclusions

Genomic data are now relatively inexpensive to collect and phenotypes remain to be the primary way to define organisms (Lehner, 2013). Many genotyping technologies exist and these technologies evolve which leads to heterogeneity of genomic data across independent experiments (Masseroli et al., 2016; Townend, 2018; Lüth et al., 2018). Similarly, phenotypic experiments, due to the high relative cost of phenotyping, usually can focus only on a set of key traits of interest. Therefore, when looking over several phenotypic datasets, the usual case is that these datasets are extremely heterogeneous and incomplete, and the data from these experiments accumulate in databases (Maiella et al., 2018; Alaux et al., 2018).

This presents a challenge but also an opportunity to make the most of genomic/phenotypic data in the future. In the long term, such databases of genotypic and phenotypic information will be invaluable to scientists as they seek to understand complex biological organisms. Issues and opportunities are beginning to emerge, like the promise of gathering phenotypical knowledge from totally independent datasets for meta-analyses.

To address the challenges of genomic and phenotypic data integration (Suravajhala et al., 2016; Stark et al., 2019), we developed a simple and efficient approach for integrating data from multiple sources. This method can be used to combine information from multiple experiments across all levels of the biological hierarchy such as microarray, gene expression, microfluidics, and proteomics will help scientists to discover new information and to develop new approaches.

For example, **Figure 8** shows that we can estimate the full genomic relationship matrix more precisely from 10 independent partially overlapping datasets of 200 genotypes and 2,000 markers each than estimating from a dataset (for the combined set of genotypes) that has 2,000 fixed markers. Twenty independent genomic datasets of 200 genotypes and 2,000 markers are as good as one genomic dataset with 5,000 markers. When we compare it to the rest of the entries, imputation is the least effective for estimating the unobserved parts of the genomic relationship matrix. This suggests that accounting for incomplete genetic relationships would be a more promising approach than estimating the genomic features by imputation and then calculating the genomic relationship matrix.

**Figure 7** shows we can accurately estimate the unobserved relationships among the genotypes in two independent pedigree-based relationship matrices by genotyping a small proportion of the genotypes in these datasets. For instance, the mean correlation for the worst-case setting (50 genotypes in each pedigree and 10 from each of the pedigree genotyped) was 0.72. This value increased up to 0.94 for the best case (250 genotypes in each pedigree and 40 from each of the pedigree genotyped).

Linear mixed modes with marker-based additive relationship matrix are the standard approach to estimate GEBVs. If the phenotypic information corresponding to the genotypes in one or more of the component matrices is missing then the genotypic value estimates can be obtained using the available phenotypic information. In this sense, the combined genomic information links all the genotypes and the experiments.

Imputation has been the preferred method when dealing with incomplete and datasets (Browning, 2008; Browning and Browning, 2009; Howie et al., 2011; Druet et al., 2014; Erbe et al., 2016). However, imputation can be inaccurate if the data is very heterogeneous (Van Buuren, 2011). In these cases, as seen in applications above, the proposed approach which uses the relationships instead of the actual features seems to outperform imputation for inferring genomic relationships. Besides, the methods introduced in this article are useful even when imputation is not feasible. For example, two partially overlapping relationship matrices, one pedigree-based and the other can be combined to make inferences about the genetic similarities of genotypes in both of these datasets (**Figure 7**).

There are also limitations to our approach. In particular when we combine data using relationship matrices original features (markers) are not imputed. Our method may not be the best option when inferences about genomic features are needed, such as in GWAS. We can address this issue by imputing the missing features using the combined relationship matrix, for instance, using a k-nearest neighbor imputation (Hastie et al., 2001) or by kernel smoothing. Moreover, if the marker data in the independent genomic studies can be mapped to local genomic regions, then the combined relationship matrices can be obtained for these genomic regions separately. Then a kernel-based model such as the ones in Yang et al. (2008); Akdemir and Jannink (2015) can be used for association testing. The nature of missingness in data will also affect our algorithm’s performance. Inference based on approaches that ignore the missing data mechanisms is valid for missing completely at random, missing at random but probably not for not missing at random (Rubin, 1976; Little and Rubin, 2002). The results of our algorithm depend on the prior information that is expressed in the initial estimate of the combined relationship matrix. This dependence, on the other hand, will decreases as the number of partial relationship matrices increases since these incomplete relationship matrices take the role of independent samples to update our prior information. When the sample size (i.e., the number of relationship matrices that are combined) is small this matrix should be carefully selected.

As it can be seen in **Figure 10B**, the genomic prediction accuracies can be low when predicting over heterogeneous populations. Nevertheless, using correlated traits in a multi-trait genomic prediction model can lead to improved prediction accuracies by borrowing information among the traits. In particular, if some unbalanced phenotypic data are available for the target set and a training set of genotypes, these can be used as additional anchors to improve accuracy. Similarly, incomplete environmental data about the different experiments in the target and training sets can be combined using the methods discussed here to possibly improve genomic prediction accuracies. The difficulty in predicting over heterogeneous populations could also be due to genetic variants are specific to particular populations. In this case, the populations could be clustered into groups and genomic prediction can be applied within each group. An alternative way to select a sub-population for training for a specific target set lies in selecting an optimized training population from a large set of candidates for that target set (Isidro et al., 2015).

### Software and Data Availability

The software was written using C++ and R and an R (R Core Team (2019) package **CovCombR** (Akdemir et al., 2020) is made available publicly. The code and data for replicating some of the analysis can be requested from the corresponding authors.

## Data Availability Statement

The datasets analyzed in this study are publicly available and can be obtained from the sources cited within.

## Author Contributions

DA: Conception and design of the work, statistics, derivations, proofs, R programs, simulations, drafting the article, and critical revision of the article. JI: R graphical programs, statistics, drafting the article, critical revision of the article. RK: Critical revision of the article.

## Funding

Results have been achieved within the framework of the first transnational joint call for research projects in the SusCrop ERA-Net Cofund on Sustainable Crop Production, with funding from the Research Council of Norway (NFR grant 299615) “Deutsches Bundesministerium für Bildung und Forschung” (031B0810), “Bundesministerium für Nachhaltigkeit und Tourismus Österreich” (Forschungsprojekt Nr. 101402), the Genome Canada project CTAG2, and the Canadian Agricultural Partnership administered by the Canadian Wheat Research Coalition, and the Department of Agriculture, Food and the Marine (DAFM), Ireland.

## Conflict of Interest

The authors declare that the research was conducted in the absence of any commercial or financial relationships that could be construed as a potential conflict of interest.

## Footnotes

1 In what follows, we will refer to genetic relationship matrices that measure how genotypes are related (See Supplementary Section 5.3 for a description of how to calculate a genetic relationship matrix from genome-wide markers [genomic relationship matrix]). However, a theme in this article is that a genetic relationship matrix is a special kind of covariance matrix. Therefore, the same arguments below apply to covariance matrices that measure the relationship between traits or features.

2 We used correlations instead of covariances because the phenotypic experiments were very heterogeneous in terms of the variances of the different traits.
