# Supplementary material for: Combining Partially Overlapping Multi-Omics Data in Databases Using Relationship Matrices
Source: Front Plant Sci. 2020 Jul 14;11:947. doi: 10.3389/fpls.2020.00947 (PMC7381228; doi:10.3389/fpls.2020.00947)
Supplement: Supplementary file 1 [file DataSheet_1.pdf]

Supplementary Materials: Combining Partially Overlapping Multi-Omics Data in Databases Using Relationship Matrices

Deniz Akdemir [1,*], Ron Knox [3] and Julio Isidro y Sánchez [1,2*]

[1] *Agriculture & Food Science Centre, Animal and Crop Science Division, University College Dublin, Dublin, Ireland*
[2] *Centro de Biotecnologia y Genómica de Plantas (CBGP, UPM-INIA)*
Universidad Politécnica de Madrid (UPM) - Instituto Nacional de Investigación y Tecnologia Agraria y Alimentaria (INIA)
Campus de Montegancedo-UPM
28223-Pozuelo de Alarcón, (Madrid), Spain
[3] *SCRDC-CRDSW, Swift Current Research and Development Centre, Swift Current, Saskatchewan, Canada*

Correspondence*:
Deniz Akdemir - Julio Isidro-Sánchez
deniz.akdemir.work@gmail.com - j.isidro@upm.es

1 SUPPLEMENTARY METHODS

1.1 Wishart EM-Algorithm

The Wishart EM-Algorithm maximizes the likelihood function for a random sample of incomplete observations from a Wishart distribution with fixed degrees of freedom since it is an EM-Algorithm (Dempster et al., 1977, 1981). To the best of our knowledge, this is the first study that derives the EM-Algorithm for the following case.

Let $G_{a_1}, G_{a_2}, \ldots, G_{a_m}$ be **independent but partial** realizations from a Wishart distribution with a known degrees of freedom $\nu > n$ and a covariance parameter $\Psi = \Sigma/\nu$, where Σ is the covariance matrix for a set K of n variables and the subindices $a_1, a_2, \ldots, a_m$ denote different subsets of the set K. We want to estimate the overall covariance matrix Σ using $G_{a_1}, G_{a_2}, \ldots, G_{a_m}$. If we focus on a single relationship matrix G_{a_i} we drop the subscript and write G_a We write $\bar{G}_a$ for the completed version of G_a obtained by complementing each of the observed data G_a with the missing data components $G_B = (G_{ab}, G_b)$ and assume $\bar{G}_a$ is partitioned as

$$\begin{bmatrix} G_a & G_{ab} \\ G_{ba} & G_b \end{bmatrix}.$$

We assume a similar partitioning of $\Psi^{(t)}$ as

$$\begin{bmatrix} \Psi_a^{(t)} & \Psi_{ab}^{(t)} \\ \Psi_{ba}^{(t)} & \Psi_b^{(t)} \end{bmatrix}$$

where $\Psi_a^{(t)}$ is the part of matrix that correspond to the variables a, $\Psi_b^{(t)}$ is the part of matrix that correspond to the variables b, and $\Psi_{ab}^{(t)} = \Psi'^{(t)}_{ba}$ is the part that correspond to the covariance of variables in a and b. Expectation of each G_a is therefore equal to Σ_a.

The likelihood function for the observed data can be written as

$$L(\Psi|\nu, G_{a_1}, G_{a_2}, \dots, G_{a_m}) = \prod_{i=1}^{m} W(G_{a_i}|\nu, \Sigma_{a_i})$$
$$= \prod_{i=1}^{m} \frac{|G_{a_i}|^{(\nu-k_i-1)/2} \exp(-\frac{1}{2}tr(\Psi^{-1}G_{a_i}))}{\left(2^{\nu k_i/2}\pi^{k_i(k_i-1)/4} \prod_{j=1}^{k_i} \Gamma(\frac{\nu+1-j}{2})\right) |\Psi_{a_i}|^{\nu/2}}$$

The log-likelihood function with the constant terms combined in c is given by

$$l(\Psi|\nu, G_{a_1a_1}, G_{a_2a_2}, \dots, G_{a_ma_m}) = c - \frac{1}{2}\sum_{i=1}^{m} \left[tr(\Psi_{a_i}^{-1}G_{a_i})) + \nu log|\Psi_{a_i}|\right].$$

We can write the log-likelihood for the complete data up to a constant term as follows:

$$\ell^c(\Psi|\nu, G_{a_1}, G_{a_2}, \dots, G_{a_m}, G_{B_1}, G_{B_2}, \dots, G_{B_m})$$
$$= \frac{v-n-1}{2}(\sum_{i=1}^{m} log|G_{a_i}|$$
$$+\sum_{i=1}^{m} |G_{b_i} - G'_{ab_i}G_{a_i}^{-1}G_{ab_i}|)$$
$$-\frac{v}{2}(\sum_{i=1}^{m} log|\Psi_{a_i}|$$
$$+\sum_{i=1}^{m} log|\Psi_{b_i} - \Psi'_{ab_i}\Psi_{a_i}^{-1}\Psi_{ab_i}|)$$
$$-\frac{1}{2}tr(\Psi^{-1}\sum_{i=1}^{m} \bar{G}_{a_i})$$

The expectation step of the EM-Algorithm involves calculating the expectation of the complete data log-likelihood conditional on observed data and the value of Ψ at iteration t which we denote by $\Psi^{(t)}$.

$$E\left[\ell^c(\Psi|\nu, G_{a_1}, G_{a_2}, \ldots, G_{a_m}, G_{B_1}, G_{B_2}, \ldots, G_{B_m})|G_{a_1}, G_{a_2}, \ldots, G_{a_m}, \Psi^{(t)}\right]$$
$$= \frac{v-n-1}{2}(\sum_{i=1}^{m} log|G_{a_i}|$$
$$+\sum_{i=1}^{m} |\Psi_{b_i}^{(t)} - {\Psi^{(t)}}'_{ab_i}{\Psi^{(t)}}_{a_i}^{-1}\Psi_{ab_i}^{(t)}|)$$
$$-\frac{vm}{2}log|\Psi|$$
$$-\frac{1}{2}tr(\Psi^{-1}\sum_{i=1}^{m} E\left[\bar{G}_{a_i}|G_{a_i}, \Psi^{(t)}\right])$$

The maximization step of the EM algorithm which updates $\Psi^{(t)}$ to $\Psi^{(t+1)}$ by finding Ψ that maximizes the expected complete data log-likelihood. (Using (Anderson, 2003, Lemma 3.3.2)) The solution is given by:

$$\Psi^{(t+1)} = \frac{\sum_{i=1}^{m} E\left[\bar{G}_{a_i}|G_{a_i}, \Psi^{(t)}\right]}{vm}.$$

We need to calculate $E\left[\bar{G}_{a_i}|G_{a_i}, \Psi^{(t)}\right]$ for each i.

Firstly, $E\left[G_a|G_{a_i}, \Psi^{(t)}\right]$ is G_a. Secondly, $G_{ab}|G_{a_i}, \Psi^{(t)}$ has a matrix-variate normal distribution with mean $G_a{\Psi_a^{(t)}}^{-1}\Psi_{ab}^{(t)}$ (the covariance of the vectorized form is given by $G_a \otimes (\Psi_b^{(t)} - {\Psi^{(t)}}'_{ab}{\Psi^{(t)}}_a^{-1}\Psi_{ab}^{(t)}).$).

To calculate the expectation of G_b, note that we can write this term as $G_b = (G_b - G'_{ab}G_a^{-1}G_{ab}) + G'_{ab}G_a^{-1}G_{ab}$ The distribution of the first term is independent of G_a and G_{ab} and is a Wishart distribution with degrees of freedom $\nu - n_a$ and covariance parameter $\Psi_b^{(t)} - {\Psi^{(t)}}'_{ab}{\Psi^{(t)}}_a^{-1}\Psi_{ab}^{(t)}$. The second term is an inner product $(G_a^{-\frac{1}{2}}G_{ab})'(G_a^{-\frac{1}{2}}G_{ab})$. The distribution of $G_a^{-\frac{1}{2}}G_{ab}$ is a matrix-variate normal distribution with mean $G_a^{\frac{1}{2}}{\Psi_a^{(t)}}^{-1}\Psi_{ab}^{(t)}$ and covariance is given by $\Psi_b^{(t)} - {\Psi^{(t)}}'_{ab}{\Psi^{(t)}}_a^{-1}\Psi_{ab}^{(t)}$, I_{n_a} for the columns and rows correspondingly. The expectation of this inner-product is ${\Psi^{(t)}}'_{ab}{\Psi^{(t)}}_a^{-1}G_a + n_a(\Psi_b^{(t)} - {\Psi^{(t)}}'_{ab}{\Psi^{(t)}}_a^{-1}\Psi_{ab}^{(t)})$. Therefore, the expected value of G_b given $G_a, \Psi^{(t)}$ is ${\Psi^{(t)}}'_{ab}{\Psi^{(t)}}_a^{-1}G_a{\Psi^{(t)}}_a^{-1}{\Psi^{(t)}}_{ab} + n_a(\Psi_b^{(t)} - {\Psi^{(t)}}'_{ab}{\Psi^{(t)}}_a^{-1}\Psi_{ab}^{(t)}) + (\nu - n_a)(\Psi_b^{(t)} - {\Psi^{(t)}}'_{ab}{\Psi^{(t)}}_a^{-1}\Psi_{ab}^{(t)}) = \nu(\Psi_b^{(t)} - {\Psi^{(t)}}'_{ab}{\Psi^{(t)}}_a^{-1}\Psi_{ab}^{(t)}) + {\Psi^{(t)}}'_{ab}{\Psi^{(t)}}_a^{-1}G_a{\Psi^{(t)}}_a^{-1}{\Psi^{(t)}}_{ab}$. This leads to the update equation:

$$\Psi^{(t+1)} = \frac{1}{\nu m}\sum_{a\in A} P_a \begin{bmatrix} G_a & G_a(B_{b|a}^{(t)})' \\ B_{b|a}^{(t)}G_a & \nu\Psi_{bb|a}^{(t)} + B_{b|a}^{(t)}G_a(B_{b|a}^{(t)})' \end{bmatrix} P'_a \tag{S1}$$

where $B_{b|a}^{(t)} = \Psi_{ab}^{(t)}(\Psi_a^{(t)})^{-1}$, $\Psi_{bb|a}^{(t)} = \Psi_b^{(t)} - \Psi_{ab}^{(t)}(\Psi_a^{(t)})^{-1}\Psi_{ba}^{(t)}$, a is the set of variables in the given partial covariance matrix and b is the set difference of K and a. The matrices P_a are permutation matrices that put

each matrix in the sum in the same order. The initial value, $\Sigma^{(0)}$ is usually assumed to be an identity matrix of dimension n.

During the steps of the Wishart EM-Algorithm, we might encounter a matrix Ψ which is not positive definite. There are two strategies to deal with this case: 1) allow Ψ to be nondefinite but replace it with a near positive definite matrix after last iteration, 2) force Ψ to be positive definite at each iteration by replacing it with a near positive definite matrix. We have used the second approach in our implementations.

Asymptotic standard errors

Once the maximizer of $l(\Psi)$, $\widehat{\Psi}$, has been found, the asymptotic standard errors can be calculated from the information matrix of Ψ evaluated at $\widehat{\Psi}$. The log-likelihood is given by:

$$l(\Psi) = c - \frac{1}{2}\sum_{i=1}^{m}\left[tr(\Psi_{a_i}^{-1}G_{a_i})) + \nu log|\Psi_{a_i}|\right].$$

First derivative with respect to the jkth element of Ψ is given by

$$\frac{\partial l(\Psi)}{\partial\psi_{jk}} = \frac{1}{2}\sum_{i=1}^{m}\left[tr(\Psi_{a_i}^{-1}\frac{\partial\Psi_{a_i}}{\partial\psi_{jk}}\Psi_{a_i}^{-1}G_{a_i}) - \nu tr(\Psi_{a_i}^{-1}\frac{\partial\Psi_{a_i}}{\partial\psi_{jk}})\right]$$

The derivative of the above with respect to the lhth element of Ψ is given by

$$\frac{\partial^2 l(\Psi)}{\partial\psi_{jk}\partial\psi_{lh}} = \frac{1}{2}\sum_{i=1}^{m}\left[(-2tr(\Psi_{a_i}^{-1}\frac{\partial\Psi_{a_i}}{\partial\psi_{jk}}\Psi_{a_i}^{-1}\frac{\partial\Psi_{a_i}}{\partial\psi_{lh}}\Psi_{a_i}^{-1}G_{a_i}) + \nu tr(\Psi_{a_i}^{-1}\frac{\partial\Psi_{a_i}}{\partial\psi_{jk}}\Psi_{a_i}^{-1}\frac{\partial\Psi_{a_i}}{\partial\psi_{lh}})\right]$$

The expected value of the second derivative is given by

$$E(\frac{\partial^2 l(\Psi)}{\partial\psi_{jk}\partial\psi_{lh}}|\Psi = \widehat{\Psi})$$

$$= \frac{1}{2}\sum_{i=1}^{m}\left[(-2tr(\widehat{\Psi}_{a_i}^{-1}\frac{\partial\Psi_{a_i}}{\partial\psi_{jk}}\widehat{\Psi}_{a_i}^{-1}\frac{\partial\Psi_{a_i}}{\partial\psi_{lh}}\widehat{\Psi}_{a_i}^{-1}E(G_{a_i}|\Psi = \widehat{\Psi})) + \nu tr(\widehat{\Psi}_{a_i}^{-1}\frac{\partial\Psi_{a_i}}{\partial\psi_{jk}}\widehat{\Psi}_{a_i}^{-1}\frac{\partial\Psi_{a_i}}{\partial\psi_{lh}})\right]$$

$$= -\frac{v}{2}\sum_{i=1}^{m}\left[tr(\widehat{\Psi}_{a_i}^{-1}\frac{\partial\Psi_{a_i}}{\partial\psi_{jk}}\widehat{\Psi}_{a_i}^{-1}\frac{\partial\Psi_{a_i}}{\partial\psi_{lh}})\right]$$

Therefore, the information matrix is given by

$$\{I(\Psi)\}_{jk,lh} = \{-E(\frac{\partial^2 l(\Psi)}{\partial\psi_{jk}\partial\psi_{lh}}|\Psi = \widehat{\Psi})\}_{jk,lh} = \frac{v}{2}\sum_{i=1}^{m}\left[tr(\widehat{\Psi}_{a_i}^{-1}\frac{\partial\Psi_{a_i}}{\partial\psi_{jk}}\widehat{\Psi}_{a_i}^{-1}\frac{\partial\Psi_{a_i}}{\partial\psi_{lh}})\right]$$

1.2 Some Properties of Matrix Normal and Wishart Distribution

The following results and their derivations are given in classic multivariate statistics textbooks such as (Anderson, 2003) and (Gupta and Nagar, 2000; Kollo and von Rosen, 2006) and are used in the derivation of the Wishart EM-Algorithm.

- (Kollo and von Rosen, 2006, Theorem 2.2.9) Let $X \sim N_{p,n}(M, \Sigma, \Psi)$. Then, $E[XAX'] = tr(\Psi A)\Sigma + MAM'$.
- (Kollo and von Rosen, 2006, Theorem 2.4.12.)Let $G \sim W_n(\nu, \Psi)$ with Ψ and $\nu > n$.
  - Density
$$p(G) = \mathcal{W}_\nu(G|\Psi) = \frac{|G|^{(\nu-k-1)/2}\exp(-\frac{1}{2}tr(\Psi^{-1}G))}{2^{\nu k/2}\pi^{k(k-1)/4}\prod_{i=1}^{k}\Gamma(\frac{\nu+1-i}{2}))|\Psi|^{nu/2}}$$
  - $E(G) = \nu\Psi$
  - $G_{1|2}$ is independent of (G_{12}, G_{22});
  - $G_{22} \sim W_q(\nu, \Psi_{22})$;
  - The conditional distribution of G_{12} given G_{22} is multivariate Gaussian $N_{(n-q)\times q}(\Psi_{12}\Psi_{22}^{-1}G_{22}, \Lambda)$ where $\Lambda_{ij,kl} = Cov(G_{ij}, G_{kl}|G_{22}) = \Psi_{ik}^{1|2}G_{jl}$.

1.3 Genomic features, distances and kernel matrices

Let M be the $n \times m$ matrix of biallelic marker allele dosages for n genotypes and m markers, and let $n < m$. The vector of estimates of allele probabilities is given by $\boldsymbol{p}'_m = (\mathbf{1}'_n M)/(2n)$. Let $X_m = (M - 2\mathbf{1}_n\boldsymbol{p}'_m)/\sqrt{c_m}$ be the feature matrix where $c_m = 2\sum_{i=1}^{m} p_{m_i}(1 - p_{m_i})$. An additive relationship matrix can be written as $= X_m X'_m$ (VanRaden, 2008). This matrix is singular as $n < m$.

A similar relationship matrix that is nonsingular can be obtained by changing the centering and scaling of the allele dosages matrix. Let $\boldsymbol{p}_n = (\mathbf{1}'_m M')/(2m)$. Let $X = (M - 2\boldsymbol{p}_n\mathbf{1}'_m)/\sqrt{c} = M(I_n - \mathbf{1}_n\mathbf{1}'_n/n)/\sqrt{c}$ be the feature matrix where $c = \frac{1}{n}\sum_{i=1}^{n}\sum_{j=1}^{m} X_{ij}^2$. X is the row centered feature matrix scaled by the mean square root of total average heterozygosity for the genotypes. We also use the notation $G_A(X) = XX'$ and note that $G_A(X)$ can be calculated from by covariance matrix for the genotypes of the marker allele dosages matrix M by dividing it by the mean of its diagonal elements (abusing notation, this can be expressed as $G_A(X) = cov(M')/mean(diag(cov(M')))$.). This matrix is non-singular whenever the number of independent features in the data is larger than the sample size. The mean of the diagonals of this relationship matrix is one. More importantly, the same formulation applies to all types of genomic features. For instance, we can use the same formulation for marker data with higher ploidy levels, or with other forms of genomic data such as the expression data.

For each pair of genotypes $((i, j) : i, j \in (1, 2, \ldots, n))$ in M, the squared Euclidean distance using the corresponding a feature matrix $X = (\boldsymbol{x}_1, \boldsymbol{x}_2, \ldots, \boldsymbol{x}_n)'$ can be written as

$$d_{ij} = (\boldsymbol{x}_i - \boldsymbol{x}_j)'(\boldsymbol{x}_i - \boldsymbol{x}_j) = \boldsymbol{x}'_i\boldsymbol{x}_i + \boldsymbol{x}'_j\boldsymbol{x}_j - 2\boldsymbol{x}'_i\boldsymbol{x}_j = (G_A)_{ii} + (G_A)_{jj} - 2(G_A)_{ij}.$$

The squared distance matrix is defined by $D(X) = (d_{ij})$ and can be calculated from the additive relationship matrix $G_A(X) = XX'$ as

$$\begin{aligned} D(X) &= \mathbf{1}_n diag(XX')' + diag(XX')\mathbf{1}'_n - 2XX' \\ &= \mathbf{1}_n diag(G_A)' + diag(G_A)\mathbf{1}'_n - 2G_A \end{aligned}$$

Moreover, since $\mathbf{1}'X = \mathbf{0}$ and $(I - \frac{\mathbf{1}\mathbf{1}'}{n})\mathbf{1} = \mathbf{1} - \mathbf{1}\frac{\mathbf{1}'\mathbf{1}}{n} = \mathbf{1} - \mathbf{1}\frac{n}{n} = 0$, we have

$$(I - \frac{\mathbf{1}\mathbf{1}'}{n})D(X)(I - \frac{\mathbf{1}\mathbf{1}'}{n})$$
$$= (I - \frac{\mathbf{1}\mathbf{1}'}{n})\left(\mathbf{1}_n diag(G_A)' + diag(G_A)\mathbf{1}_n' - 2G_A\right)(I - \frac{\mathbf{1}\mathbf{1}'}{n})$$
$$= -2XX' = 2G_A.$$

Therefore, given $D(X)$ and letting $P = (I - \frac{\mathbf{1}\mathbf{1}'}{n})$ the additive relationship matrix can also be calculated by

$$G_A = -\frac{1}{2}PDP.$$

The genomic relationship matrices need not be additive. RKHS regression extends additive relationship based SPMMs by allowing a wide variety of kernel matrices, not necessarily additive in the input variables, calculated using a variety of kernel functions. A kernel function, $k(.,.)$ maps a pair of input points $\boldsymbol{x}$ and $\boldsymbol{x}'$ into real numbers. It is by definition symmetric ($k(\boldsymbol{x}, \boldsymbol{x}') = k(\boldsymbol{x}', \boldsymbol{x})$) and non-negative. Given the inputs for the n genotypes we can compute a kernel matrix G whose entries are $G_{ij} = k(\boldsymbol{x}_i, \boldsymbol{x}_j)$. The linear kernel function is given by $k(\boldsymbol{x}; \boldsymbol{y}) = \boldsymbol{x}'\boldsymbol{y}$. The polynomial kernel function is given by $k(\boldsymbol{x}; \boldsymbol{y}) = (\boldsymbol{x}'\boldsymbol{y} + c)^d$ for c and $d \in R$. Finally, the Gaussian kernel function is given by $k(\boldsymbol{x}; \boldsymbol{y}) = exp(-h(\boldsymbol{x}' - \boldsymbol{y})'(\boldsymbol{x}' - \boldsymbol{y}))$ where $h > 0$. The common choices for kernel functions are the linear, polynomial, Gaussian kernel functions, though many other options are available (Schölkopf and Smola, 2005; Endelman, 2011).

The relationship between the Euclidean distance matrix and the corresponding Gaussian kernel is given by

$$G_G^h(X) = \exp(-h * D(X))$$

and

$$D(X) = -\frac{\log(G_G^h(X))}{h}.$$

An important advantage of using similarity or distance matrices over the original features is that similarity of distance matrices can be calculated for variables of different type (categorical, rank, or interval-scale data). The relationship of the feature matrix, and the additive kernel and Euclidean distance allows us to generalize the additive relationship matrix to general genomic data (not necessarily marker allele dosages).

1.4 Mixed models and genomic relationship matrices

Let's start by describing how we can use a single combined genomic data. The discussion below will be biased towards a discussion variance components / mixed modeling approach since this has a special place in quantitative genetics. Mixed models have been used as a formal way of partitioning the variability observed in traits into heritable and environmental components, it is also useful in controlling for population structure and relatedness for genome-wide association studies (GWAS). However, some of the methods that are proposed can be used in other forms of statistical analysis, for instance, for descriptive purposes or in general statistical learning.

In a mixed model, genetic information in the form of a pedigree or marker allele frequencies can be used in the form of an additive genetic similarity matrix that describes the similarity based on additive genetic

effects (GBLUP). For the $n \times 1$ response vector $\boldsymbol{y}$, the GBLUP model can be expressed as

$$\boldsymbol{y} = X\beta + Z\boldsymbol{u} + \boldsymbol{e} \tag{S2}$$

where X is the $n \times p$ design matrix for the fixed effects, β is a $p \times 1$ vector of fixed effect coefficients, Z is the $n \times q$ design matrix for the random effects; the vector random effects $(\boldsymbol{u}', \boldsymbol{e}')'$ is assumed to follow a multivariate normal (MVN) distribution with mean $\mathbf{0}$ and covariance

$$\begin{pmatrix} \sigma_g^2 G & \mathbf{0} \\ \mathbf{0} & \sigma_e^2 I_n \end{pmatrix} \tag{S3}$$

where G is the $q \times q$ additive genetic similarity matrix. In this model, the labels of the genotypes (that are listed in the rows and columns of the relationship matrix G) define a factor variable with levels equal to the labels. The matrix Z is the design matrix that links the observed response in the experiment to these levels. The model (S2) is equivalent to a MM in which the additive marker effects are estimated via the following model (rr-BLUP),

$$\boldsymbol{y} = X\boldsymbol{\beta} + ZM\boldsymbol{u} + \boldsymbol{e} \tag{S4}$$

where X is the $n \times p$ design matrix for the fixed effects, β is a $p \times 1$ vector of fixed effect coefficients, Z is the $n \times q$ design matrix for the random effects M is $q \times m$ marker allele frequency centered incidence matrix; $(\boldsymbol{u}', \boldsymbol{e}')'$ follows a MVN distribution with mean $\mathbf{0}$ and covariance

$$\begin{pmatrix} \sigma_u^2 I_m & \mathbf{0} \\ \mathbf{0} & \sigma_e^2 I_n \end{pmatrix}.$$

Note that the scale of the genomic relationship matrix is irrelevant for genomic prediction or family structure correction in mixed model-based association studies. However, this quantity is important for the calculation of narrow-sense heritability. In this case, setting the average of the diagonals of the relationship makes it, in a way, compatible with the broad sense heritability calculations based on an identity relationship matrix for genotypes that already has a mean of its diagonal elements equal to one. In addition, the standard formulations of the marker-based additive matrix models used in the literature can be generalized to incorporate more complex genetic and environmental covariates.

2 SUPPLEMENTARY APPLICATIONS

2.1 Experiments with simulated data

Supplemenatry Application 1- Simulation study: Inferring the combined covariance matrix from its parts

To establish that a combined relationship can be inferred from realizations of its parts, we have conducted the following simulation study: In each round of the simulation, the true parameter value of the genomic relationship matrix was generated as $\Sigma = diag(r_1, r_2, \ldots, r_{N_{Total}}) + .3 * \mathbf{1}_{N_{Total} \times N_{Total}}$ where r_i were independently generated as $1 + .7 * u_i$ with u_i a realization from the uniform distribution over $(0, 1)$. Σ was then adjusted by dividing it with the mean value of its diagonal elements. This parameter was taken as the covariance parameter of a Wishart distribution with degrees of freedom 300 and N_{kernel} samples from this distribution are generated. After that, each of the realized relationship matrices was made partial by leaving a random sample of 10 to 40 (this number was also selected from the discrete uniform distribution for integers 10 to 40) genotypes in it. These partial kernel matrices were combined using the

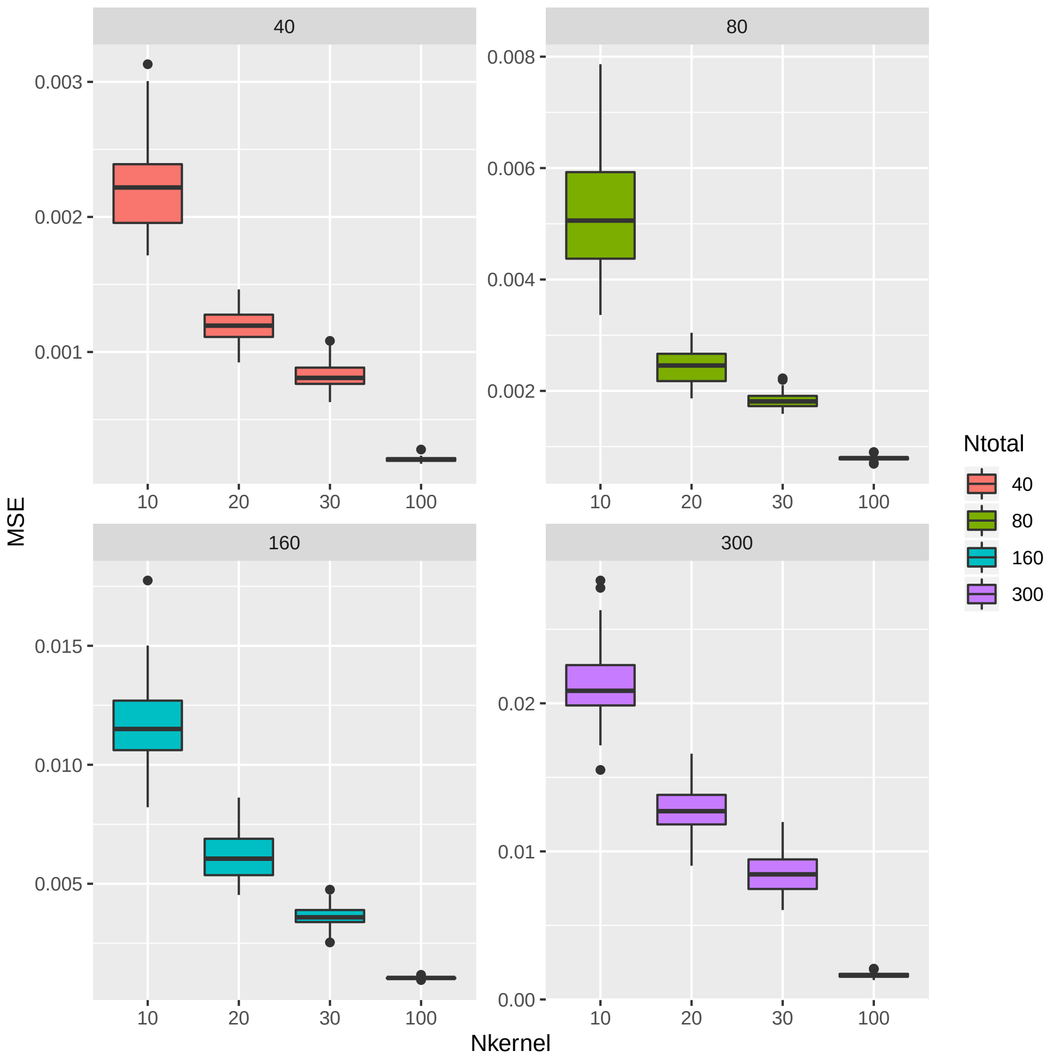

Figure S1. Application 1 - MSE's for estimating correlation parameters based on partial samples for $N_{Total} \in \{40, 80, 150, 300\}$ (number of variables in the covariance matrix) and $N_{kernel} \in \{40, 80, 150, 300\}$ (number of incomplete covariance matrix samples). Each incomplete covariance matrix was had a random size between 10 to 40. The MSE's are calculated over 10 replications of the experiment.

Wishart EM-Algorithm iterated for 50 rounds (each round cycles through the partial relationship matrices in random order). The resultant combined relationship matrix $\widehat{\Sigma}$ was compared with the corresponding parts of the parameter Σ[1] by calculating the mean squared error between the upper diagonal elements of these matrices. This experiment was replicated 10 times for each value of $N_{Total} \in \{40, 80, 150, 300\}$ and $N_{kernel} \in \{40, 80, 150, 300\}$.

The results of this simulation study are summarized in Figure S1. For each covariance size, the MSE's decreased as the number of incomplete samples increased. On the other hand, as the size of the covariance matrix increased the MSEs increased.

Supplementary Application 2- Simulation study: Likelihood Convergence

[1] In certain instances, the union of the genotypes in the parts did not recover all of the N_{Total} genotypes, therefore this calculation was based on the recovered part of the full genomic relationship matrix

The Wishart EM-Algorithm maximizes the likelihood function for a random sample of incomplete observations from a Wishart distribution. The derivation of this feature is given in the Supplementary. In this application, we explore the convergence of the algorithm for several instances starting from several different initial estimates.

The application is composed of 10 experiments each of which starts with a slightly different assumed Wishart covariance parameter[2]. For each true assumed covariance matrix, we have generated 10 partial samples including between n_{min} and n_{max} genotypes (random at discrete uniform from n_{min} to n_{max}) each using the Wishart distribution. n, the total number of genotypes in the assumed relationship matrix was taken to be 100 or 1000. Corresponding to this two matrix sizes the $n_m in$ and $n_m ax$ are taken as 10 and 25 or 100 and 250. These 10 matrices are combined using the Wishart EM-Algorithm 10 different times each times using a slightly different initial estimate of the covariance parameter[3] . We record the path of the log-likelihood function for all these applications.

At each instance of the parameter and a particular sample, the likelihood functions converged to the same point (See Figure S2). We have not observed any abnormalities in convergence according to these graphs.

Heatmap for 95 wheat traits

Phenotypic network for 186 traits based on phenotypic correlations (Wheat, Barley, and Oat Phenotypic Trials from Triticeae Toolbox)

2.2 Supplementary Figures

[2] $\Sigma = diag(\boldsymbol{b}+1) + .2\mathbf{1}_{n\times n}$ where $\boldsymbol{b}_i$ for $i = 1, 2, \ldots, n$ are i.i.d. uniform between 0 and 1.

[3] $\Sigma_0 = diag(.5\boldsymbol{b}+1) + .3 * b_0\mathbf{1}_{n\times n}$ where $\boldsymbol{b}_i$ for $i = 0, 2, \ldots, n$ are i.i.d. uniform between 0 and 1.

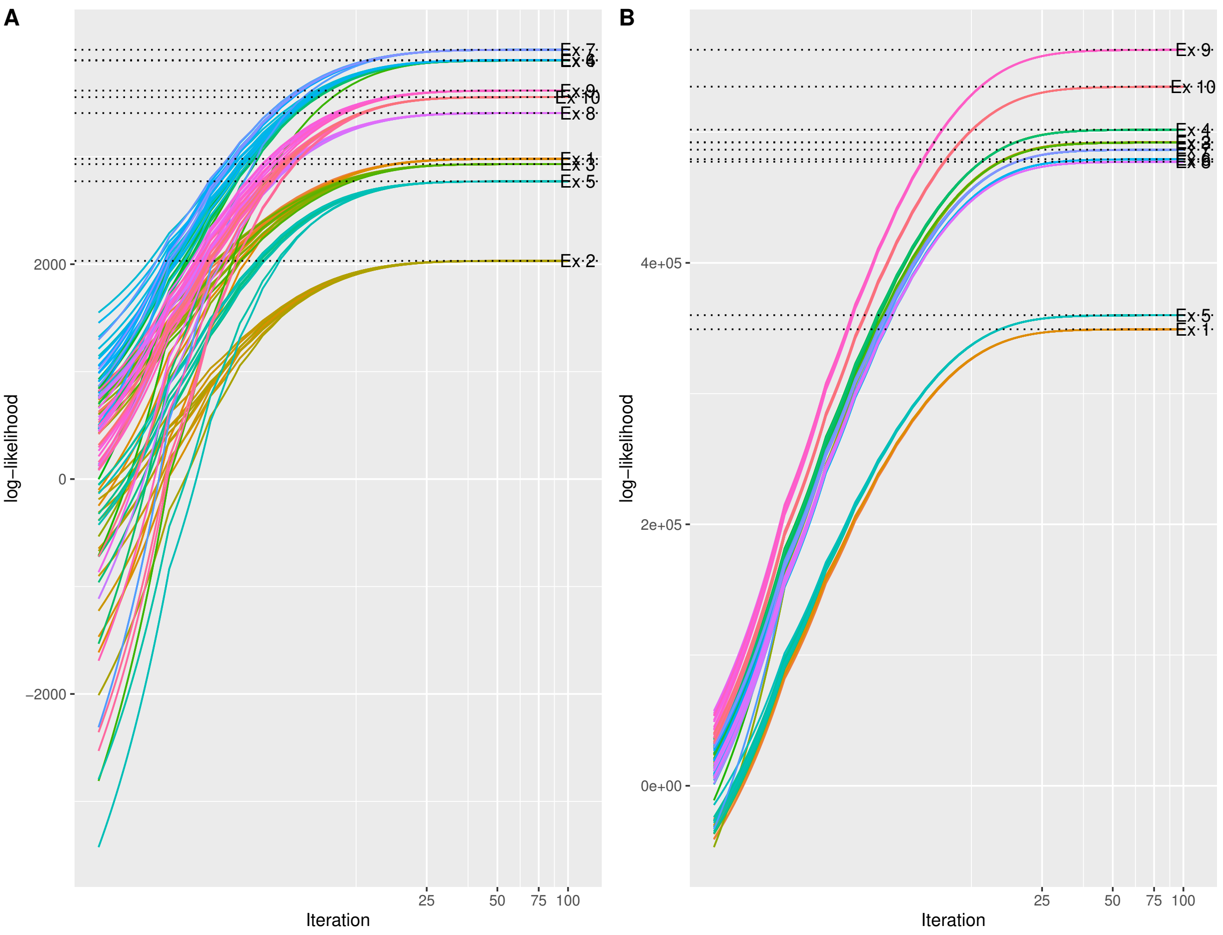

Figure S2. Application 2 - Convergence of log-likelihood function: Each color represents a different experiment. In each experiment, a sample of incomplete covariance matrices from a Wishart distribution was combined using the Wishart EM-Algorithm starting from 10 different slightly different random initial estimates. n, the total number of genotypes in the assumed relationship matrix was taken to be 100 (**A**) or 1000 (**B**).

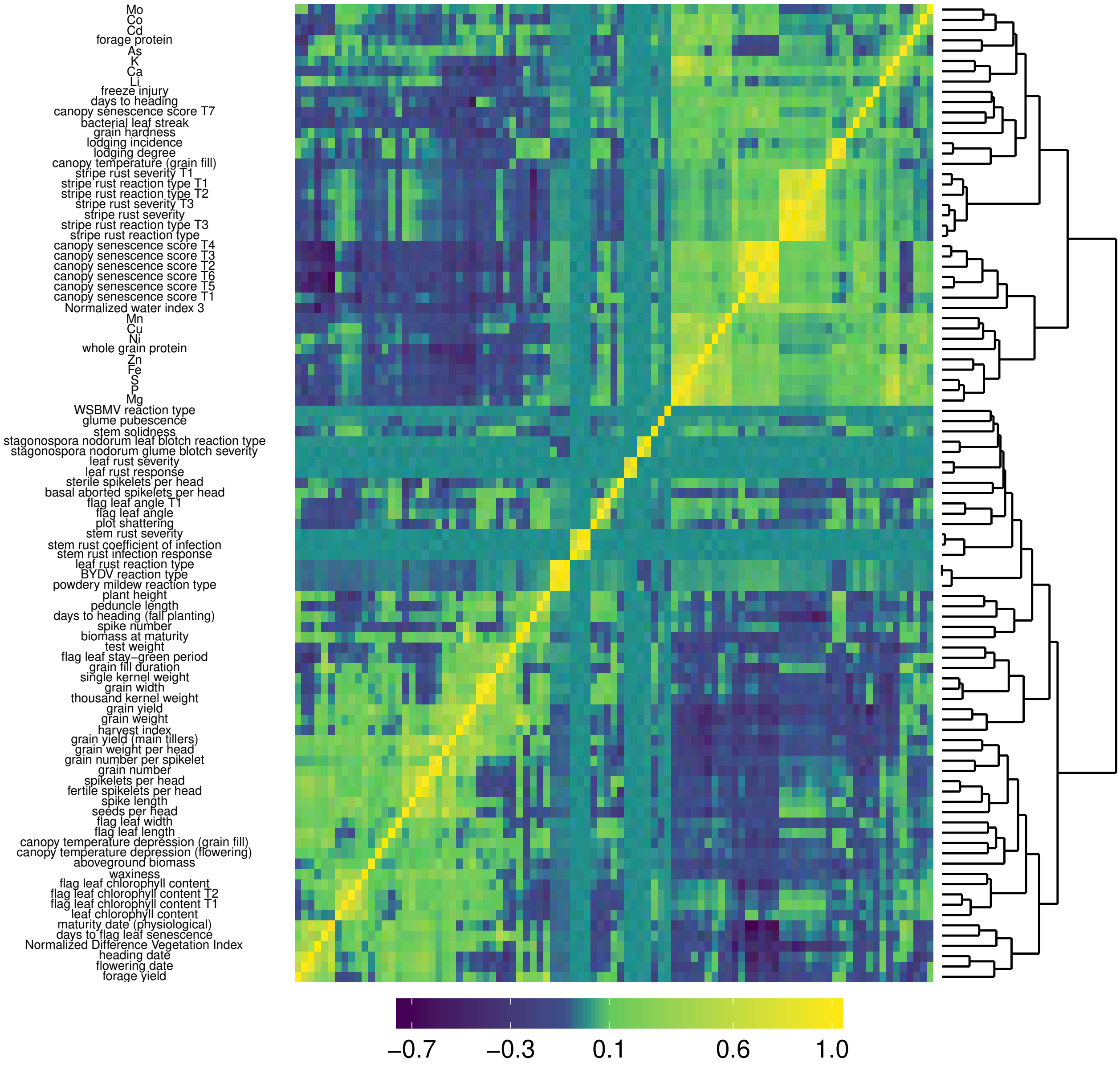

Figure S3. Triticeae dataset: Combining the phenotypic correlation matrices from 144 wheat datasets covering 95 traits. Clustered heatmap of Pearson correlation coefficients provides a global overview of phenotypic correlation across wheat traits. Yellow denotes high correlation, dark green high anti-correlation.

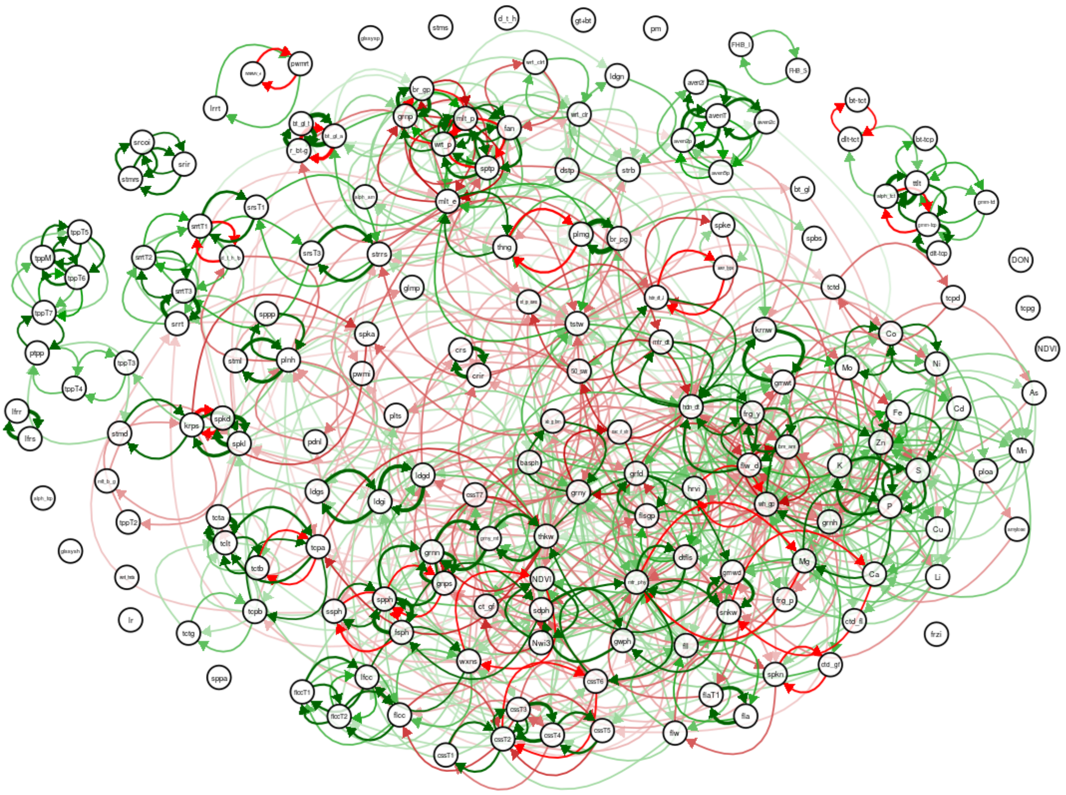

name	label	name	label	name	label	name	label
aboveground biomass	Above_B	fertile spikelets per head	FS	harvest index	HI	seeds per head	SPH
As	As	flag leaf angle	FLA	heading date	Heading date	single kernel weight	SKW
bacterial leaf streak	BLS	flag leaf angle T1	FLAT1	K	K	spike length	Slength
basal aborted spikelets per head	BAS	flag leaf chlorophyll content	FLCC	leaf chlorophyll content	LCC	spike number	SN
biomass at maturity	Biomass M	flag leaf chlorophyll content T1	FLCCT1	leaf rust reaction type	LRRT	spikelets per head	N_Spikeletes
Ca	Ca	flag leaf chlorophyll content T2	FLCCT2	leaf rust response	LRR	stem rust coefficient of infection	SRCI
canopy senescence score T1	CST1	flag leaf length	FL length	leaf rust severity	LRS	stem rust infection response	SRIR
canopy senescence score T2	CST2	flag leaf stay-green period	Stay green	Li	Li	stem rust severity	SRV
canopy senescence score T3	CST3	flag leaf width	FL width	lodging degree	L_d	stem solidness	Stem solidness
canopy senescence score T4	CST4	flowering date	FD	lodging incidence	Lodging I	sterile spikelets per head	SS
canopy senescence score T5	CST5	forage protein	FP	maturity date (physiological)	Maturity	stripe rust reaction type	SRRT
canopy senescence score T6	CST6	forage yield	FY	Mg	Mg	stripe rust reaction type T1	SRRT1
canopy senescence score T7	CST7	freeze injury	FI	Mn	Mn	stripe rust reaction type T2	SRRT2
canopy temperature (grain fill)	CT	glume pubescence	GP	Mo	Mo	stripe rust reaction type T3	SRRT3
canopy temperature depression (flowering)	CTDF	grain fill duration	GFD	Ni	Ni	stripe rust severity	SRS
canopy temperature depression (grain fill)	CTDGF	NA	NA	Normalized Difference Vegetation Index	NDVI	stripe rust severity T1	SRST1
Cd	Cd	grain number	GN	Normalized water index 3	NWI3	stripe rust severity T3	SRST3
Co	Co	grain number per spikelet	NGSpikelet	P	P	test weight	TW
Cu	Cu	grain weight	GW	peduncle length	PL	thousand kernel weight	TKW
days to flag leaf senescence	DTFLS	grain weight per head	GWH	plant height	P.Height	waxiness	Waxiness
days to heading	DTH	grain width	GW	plot shattering	PS	whole grain protein	WGP
days to heading (fall planting)	DTH2	grain yield	G.Yield	powdery mildew reaction type	PM	WSBMV reaction type	WSBMV_RT
Fe	Fe	grain yield (main tillers)	GYMT	S	S	Zn	Zn

Figure S4. Triticeae datasets: Combining the phenotypic correlation matrices from oat (78 correlation matrices), barley (143 correlation matrices) and wheat (144 matrices) datasets downloaded and selected in a similar way as in Application 5 were combined to obtain the DAG involving 196 traits.

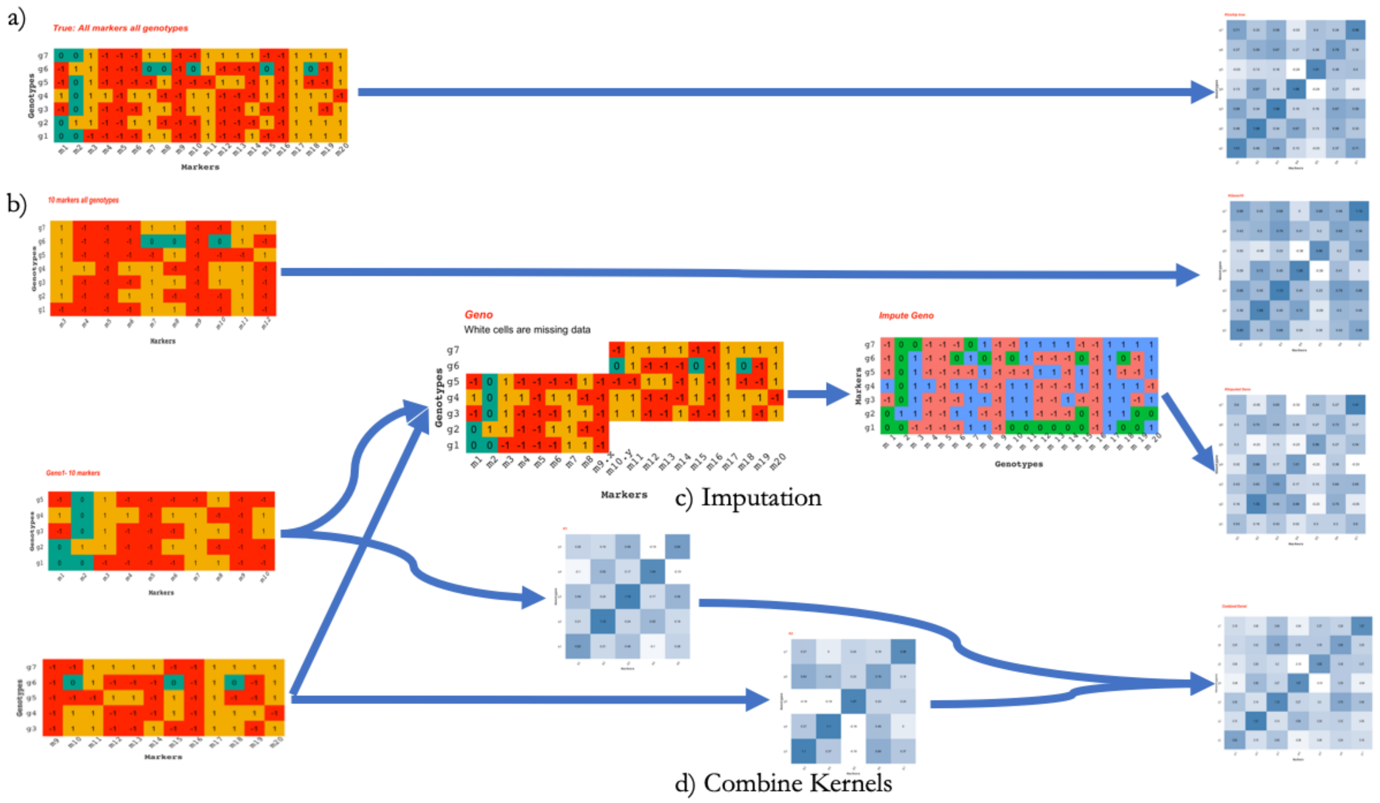

Figure S5. Pictorial representation for some of the different scenarios in Application 2 with reduced number of markers, genotypes and number of independent marker datasets. a) Assumed truth, b) All genotpes using 10 markers, c) Imputation of 2 independent marker datasets, d) Combining the relationship matrices from 2 independent marker datasets.

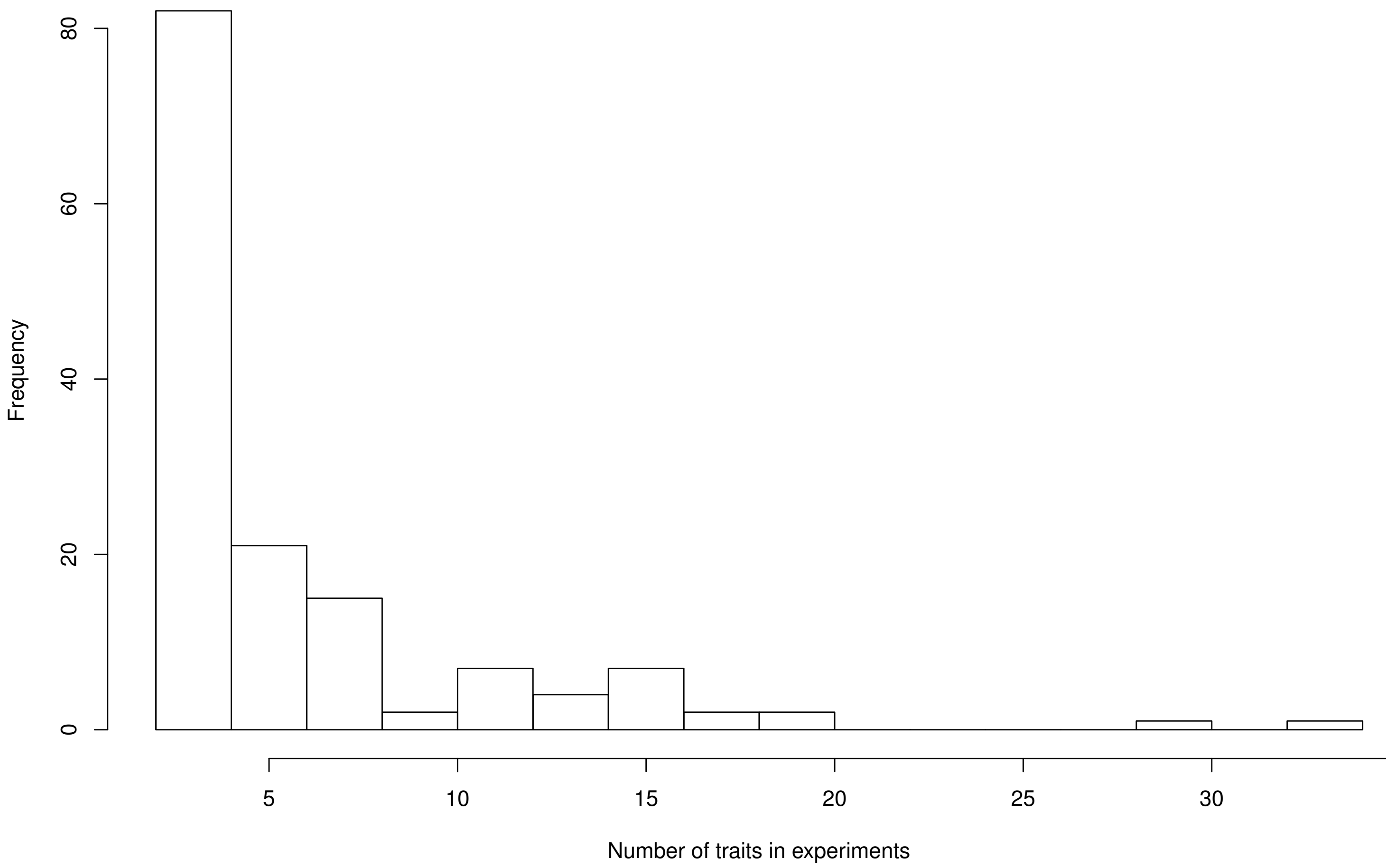

Figure S6. Triticeae dataset: The distribution of the numbers of traits in 144 phenotypic trials at Triticeae Toolbox for wheat. The mean and the median of the number of traits in these trials were 5.9 and 4 correspondingly.

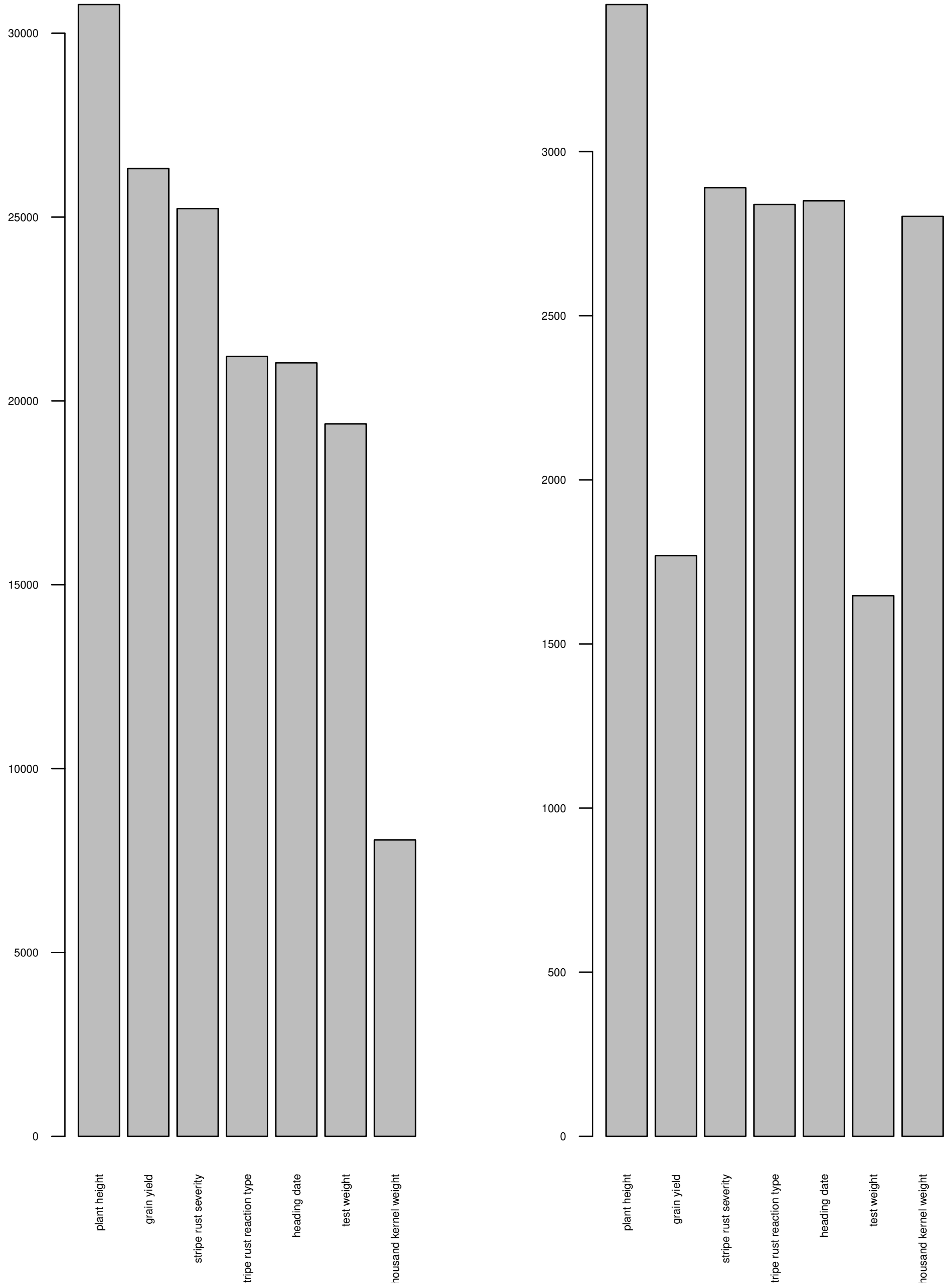

Figure S7. Triticeae dataset: Number of phenotypic observations (left) and the number of genotypes available in Triticeae Toolbox for a set of 7 selected traits for the 9102 genotyes in the combined relationship matrix.
